# Programmed cell death: molecular mechanisms, biological functions, diseases, and therapeutic targets

**DOI:** 10.1002/mco2.70024

**Published:** 2024-11-28

**Authors:** Shen'er Qian, Yao Long, Guolin Tan, Xiaoguang Li, Bo Xiang, Yongguang Tao, Zuozhong Xie, Xiaowei Zhang

**Affiliations:** ^1^ Department of Otolaryngology Head and Neck Surgery The Third Xiangya Hospital, Central South University Changsha Hunan China; ^2^ Cancer Research Institute School of Basic Medicine Central South University Changsha Hunan China; ^3^ Department of Pathology Xiangya Hospital, Central South University Changsha Hunan China; ^4^ Department of Otolaryngology Head and Neck Surgery Shanghai Ninth People's Hospital, Shanghai Jiao Tong University School of Medicine Shanghai China; ^5^ Ear Institute Shanghai Jiao Tong University School of Medicine, Shanghai Key Lab Shanghai China; ^6^ Furong Laboratory Central South University Changsha Hunan China; ^7^ Department of Otolaryngology Head and Neck Surgery The Second Xiangya Hospital Central South University Changsha Hunan China

**Keywords:** cancers, cell homeostasis, clinical transformation, neurodegenerative disorders, programmed cell death

## Abstract

Programmed cell death represents a precisely regulated and active cellular demise, governed by a complex network of specific genes and proteins. The identification of multiple forms of programmed cell death has significantly advanced the understanding of its intricate mechanisms, as demonstrated in recent studies. A thorough grasp of these processes is essential across various biological disciplines and in the study of diseases. Nonetheless, despite notable progress, the exploration of the relationship between programmed cell death and disease, as well as its clinical application, are still in a nascent stage. Therefore, further exploration of programmed cell death and the development of corresponding therapeutic methods and strategies holds substantial potential. Our review provides a detailed examination of the primary mechanisms behind apoptosis, autophagy, necroptosis, pyroptosis, and ferroptosis. Following this, the discussion delves into biological functions and diseases associated dysregulated programmed cell death. Finally, we highlight existing and potential therapeutic targets and strategies focused on cancers and neurodegenerative diseases. This review aims to summarize the latest insights on programmed cell death from mechanisms to diseases and provides a more reliable approach for clinical transformation.

## Graphical abstract

Programmed cell death is a type of autonomic and orderly cell death mode controlled by genes. We reviewed molecular mechanisms of five programmed cell death, including apoptosis, autophagy, pyroptosis, necroptosis, and ferroptosis. Programmed cell death is widely involved in the normal physiological process of the organism. We described the normal biological functions of programmed cell death from four perspectives like development and tissue homeostasis, immune regulation, response to cellular stress, and elimination of damaged or infected cells. Conversely, dysregulated PCD is implicated in the occurrence and progression of numerous diseases. Relevant progressions of programmed cell death observed in cancer, neurodegenerative diseases, and autoimmune disorders were summarized. Based on these, key proteins in each pathway thus become important regulatory targets. PCD‐based therapies, including small molecule drugs, cannot be ignored.

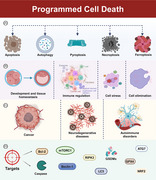

## 1 INTRODUCTION

Programmed cell death (PCD) represents an actively regulated form of cellular demise orchestrated by an array of genes.^1^ While the term “programmed cell death” was introduced in the 1960s, the detailed molecular mechanisms underlying this process continue to be a focal point of research.^2^ Recent studies have revealed multiple patterns of PCD. The main representatives are apoptosis, autophagy, necroptosis, pyroptosis, and ferroptosis.^3^

As a foundational biological process, cell death is integral to embryogenesis and tissue homeostasis, immune regulation, response to cellular stress, and the elimination of damaged or infected cells.^4^ Beyond its role in development and physiological processes, PCD, as a highly regulated self‐destructive pathway, significantly impacts the pathogenesis of various diseases, including cancer, neurodegenerative disorders, and autoimmune conditions, where both excessive and deficient PCD contribute to disease progression.^5^ Deficient cell death is often linked to tumor formation and autoimmune disorders, while excessive cell death is a driver in neurodegeneration.^6^

The ongoing discovery of PCD mechanisms in specific pathological contexts has positioned regulation induced by human as a promising therapeutic strategy. Reflecting the clinical potential of PCD modulation, drugs like venetoclax have been approved to target PCD pathways for leukemia treatment.^7^ Comprehensive insights into PCD represent a transformative opportunity for advancing human health, underscoring the necessity to establish links between PCD and organismal health.

This review initially explores the molecular mechanisms of key PCD types, including apoptosis, autophagy, necroptosis, pyroptosis, and ferroptosis. It then addresses the biological functions of PCD as essential elements in normal human growth and development. Subsequently, recent progress on the implications of dysregulated cell death in human diseases is thoroughly reviewed, culminating in an analysis of PCD‐centered therapeutic strategies that summarize current advancements and outline future research directions.

## 2 MOLECULAR MECHANISMS OF PCD

**FIGURE 1:**
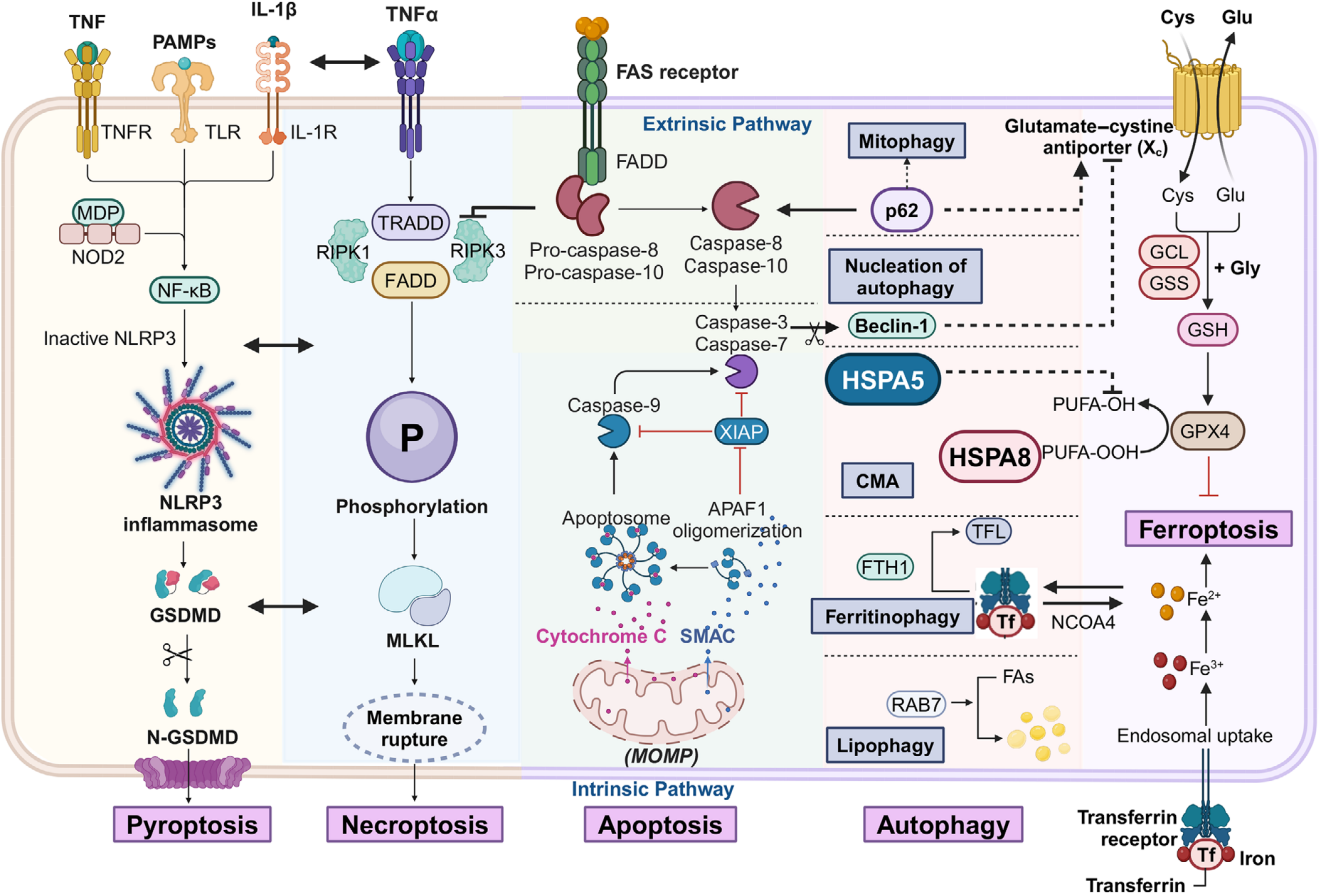
Molecular mechanisms of programmed cell death. Apoptosis is characterized by a caspase cascade that occurs without inflammatory responses and can be classified into intrinsic and extrinsic pathways. Autophagy is a highly conserved cellular catabolic mechanism that depends on lysosomal function, encompassing four distinct stages: initiation, nucleation, extension, and fusion. Both pyroptosis and necroptosis represent forms of immunogenic cell death, with caspase activity and RIPK1/3 mediating the proteolysis of GSDMx and phosphorylation of MLKL, respectively, leading to pore formation. Ferroptosis is defined by an imbalance of iron‐related lipid peroxides within the cell, a condition that arises from the complex interplay of intracellular redox reactions. MDP, muramyl dipeptide; NOD2, nucleotide‐binding oligomerization domain 2; FTH1, ferritin heavy chain 1; TFL, ferritin light chain; RAB7, Ras‐related protein Rab 7a; FAs, free fatty acids; Cys, cysteine; Glu, glutamate; GCL, glutamate cysteine ligase; GSS, glutathione synthetase; Gly, glycine; GSH, glutathione. Figures were created with biorender.com.

PCD is a highly regulated process driven by complex molecular mechanisms. Various intracellular and extracellular signals initiate specific molecular cascades that culminate in cell death. The primary mechanisms include apoptosis and autophagy, with apoptosis often regarded as the most extensively studied form of PCD. Autophagy, conversely, serves as both a survival mechanism and a form of PCD, depending on cellular context and conditions. Additionally, necroptosis, pyroptosis, and ferroptosis represent distinct and increasingly studied forms of regulated cell death. Both necroptosis and pyroptosis can trigger immune responses, contrasting with the typically immunologically silent apoptosis. Ferroptosis, uniquely, introduces metal ions as critical elements in PCD for the first time. These mechanisms are interconnected and interdependent, highlighting the importance of elucidating the molecular pathways and regulatory networks underlying PCD to deepen scientific understanding (Figure 1).

### 2.1 Apoptosis

Described initially by Kerr in 1972, apoptosis is a fundamental form of PCD.^8^ This process, defined by a caspase cascade, induces cellular demise without typically eliciting inflammatory responses. This pathway is further classified into intrinsic and extrinsic apoptosis, differentiated by their upstream triggers. Extrinsic apoptosis is initiated through the activation of death receptors on the plasma membrane, whereas intrinsic apoptosis predominantly results from cellular stress.^9^, ^10^

#### 2.1.1 Intrinsic apoptosis

The intrinsic, or mitochondrial, apoptosis pathway is initiated by shifts in intracellular signals in response to various stressors, such as DNA damage, severe oxidative stress, and growth factor deprivation.^11^ These stressors disrupt the balance between prosurvival and proapoptotic factors within the B cell lymphoma 2 (BCL‐2) protein family. BCL‐2, a mammalian homolog of the CED‐9 gene in *C. elegans*, was identified in 1985, and numerous homologous proteins have since been recognized as critical regulators in intrinsic apoptosis. The proapoptotic roles of BCL‐2 associated X protein (BAX) and BCL‐2 antagonist/killer (BAK) depend on their binding interactions, which antiapoptotic BCL‐2 family members can disrupt to regulate apoptosis.^12^ When BAX and BAK are activated, they mediate mitochondrial outer membrane permeabilization (MOMP) with assistance from BH3‐only proteins such as BCL‐2 homology 3 interacting domain death agonist (BID) and BCL‐XL/BCL‐2 associated death promoter (BAD). These proteins employ their BCL‐2 homology 3 (BH3) domains to neutralize antiapoptotic BCL‐2 proteins, including BCL‐2 and BCL‐XL, and some BH3‐only proteins can directly activate BAX and BAK.^13^

Increased mitochondrial membrane permeability induces a significant influx of Ca^2+^, promoting the release of proapoptotic factors like cytochrome *C* and second mitochondria‐derived activator of caspases (SMAC).^14^ Cytochrome *C* binding to apoptotic protease activating factor‐1 (APAF‐1) induces a conformational change, facilitating apoptosome assembly and the subsequent recruitment and activation of procaspase‐9.^15^ The apoptotic caspases are categorized into initiator and effector caspases. Cleavage by caspase‐9 activates downstream effectors, such as caspase‐3 and caspase‐7, which initiate a cascade of proteolytic events that dismantle cellular structures.^16^ For instance, caspase‐3 and caspase‐7 cleave the inhibitor of caspase‐activated deoxyribonuclease, allowing caspase‐activated deoxyribonuclease to fragment DNA, thereby enhancing apoptotic efficiency.^17^ SMAC, or direct inhibitor of apoptosis protein (IAP) binding protein with low PI, further promotes apoptosis by targeting the degradation of IAPs,^18^ a family of proteins that regulate apoptosis levels, with X‐linked inhibitor of apoptosis protein (XIAP directly inhibiting caspase‐3 and caspase‐7.^19^ Emerging evidence suggests revising the conventional linear caspase cascade to a more interconnected circular model; the inhibition of mitochondria‐dependent apoptosis observed in caspase‐3 and caspase‐7 knockout mice substantiates this nonlinear perspective.^20^

#### 2.1.2 Extrinsic apoptosis

The extrinsic apoptosis pathway is initiated by caspase‐8, which subsequently activates the executioner caspases, caspase‐3 and caspase‐7. Upon binding of exogenous death ligands to death receptors on the cell membrane, primary apoptotic signals are conveyed.^21^ Major ligand–receptor pairs in this pathway include factor related apoptosis ligand (FASL) with factor related apoptosis (FAS), tumor necrosis factor (TNF) with tumor necrosis factor receptor 1 (TNFR1), and tumor necrosis factor related apoptosis inducing ligand (TRAIL) with TRAIL‐R1 or TRAIL‐R2 (DR4/DR5).^22^ Activation of death receptors promotes the recruitment of TNFRSF1A associated via death domain (TRADD), FAS associated via death domain (FADD), and caspase‐8/10, assembling the death‐inducing signaling complex.^23^ The activation of caspase‐3 and caspase‐7, essential for executing apoptosis, is mediated through caspase‐8 cleavage, analogous to caspase‐9′s role in the intrinsic pathway. The specific function of caspase‐10, however, remains unclear.

TNFR signaling extends beyond cell death, also influencing inflammation and cell survival across various contexts. Upon activation, TNFR recruits receptor‐interacting protein kinase 1 (RIPK1) and cellular inhibitors of apoptosis proteins (cIAPs) through TRADD's interaction with TNF receptor‐associated factor 2, forming complex I.^24^ The cIAPs facilitate RIPK1 modification via Lys63‐linked ubiquitination, enhancing recruitment of canonical IkB kinase complexes and contributing to NF‐κB pathway activation.^25^ However, functional inhibition of complex I components, such as cIAPs, transforming growth factor β activated kinase 1 (TAK1), or Tank‐binding kinase 1 (TBK1), results in the transition to complex II, or ripoptosome, composed of RIPK1, FADD, caspase‐8, and cFLIP.^26^ Here, the balance of cFLIP and caspase‐8 is pivotal, with cFLIP acting as a suppressor to limit caspase‐8 activation by TRADD and FADD. Consequently, ripoptosome exhibits proapoptotic activity only under conditions of low cFLIP levels.^27^ In cases where caspase‐8 is inhibited, cell fate shifts toward necroptosis due to the absence of RIPK1 cleavage (details on this are further discussed in the necroptosis section).^28^ Caspase‐8 activation also leads to BID cleavage, generating tBID, which promotes SMAC release via MOMP. This process counteracts XIAP's inhibitory effects on apoptosis, thus establishing a critical link between the intrinsic and extrinsic apoptosis pathways.^29^

### 2.2 Autophagy

Autophagy is a highly conserved catabolic mechanism that uses lysosomes to efficiently degrade and recycle damaged, aged, or excess biomacromolecules and organelles, releasing small molecules for cellular reuse. This process is essential for maintaining cellular homeostasis under normal physiological conditions and functions as a self‐preservation mechanism under external stressors, nutrient deprivation, hypoxia, or endoplasmic reticulum (ER) stress.^30^ Autophagy is classified into three types: macroautophagy, microautophagy, and chaperone‐mediated autophagy (CMA).^31^ In microautophagy, cytoplasmic organelles or vesicles directly interact and fuse with lysosomes, displaying greater specificity than macroautophagy. CMA depends on cytoplasmic chaperones for cargo recognition, facilitating lysosomal fusion via substrate proteins and LAMP‐2A. Current research primarily focuses on macroautophagy, distinguished by its initial sequestration of target cargo within double‐membrane vesicles before lysosomal fusion. Hereafter, the term “autophagy” refers specifically to macroautophagy.^32^, ^33^, ^34^

Autophagy proceeds through four stages of cellular membrane reorganization: initiation, nucleation, extension, and fusion.^35^ The ULK1/2–ATG13–FIP200 complex is a pivotal structural entity initiating autophagy, regardless of nutrient levels.^36^ Regulation of this complex by mammalian target of rapamycin (mTOR), the mechanistic target of rapamycin, is modulated by nutrient availability; in nutrient‐rich states, mTORC1 binds to and inactivates the complex by phosphorylating unc‐51 like autophagy activating kinase 1/2 (ULK1/2) and ATG13. Nutrient deprivation lifts this inhibition by releasing mTORC1.^37^ Under normal conditions, cells maintain a basal level of autophagy due to adequate energy availability. However, during energy scarcity, adenosine 5' monophosphate activated protein kinase (AMPK) can activate autophagy by directly associating with the ULK1 complex, indicating cross‐regulation between mTORC1 and AMPK.^38^

The nucleation phase in autophagy depends on the formation of the phosphoinositide‐3‐kinase class 3 (PIK3C3) complex, stimulated by the initiation complex and primarily composed of vacuolar protein sorting 34(Vps34), Beclin‐1, ATG14, and p150.^39^ Beclin‐1 serves as a critical regulator of nucleation and can be inhibited by Bcl‐2.^40^ Vps34 catalyzes the extensive production of PtdIns3P, establishing a platform for the recruitment of autophagy‐related proteins with PI3P‐binding domains, such as WD repeat domain phosphoinositide‐interacting protein (WIPI).^41^ During nucleation, a liposome‐like membrane forms within the cytoplasm, gradually expanding into a flat structure called the phagophore, a key marker of autophagy. The phagophore continues to extend, encapsulating cytoplasmic components, including organelles, before sealing off into an autophagosome—another hallmark of autophagy, indicating the transition to the third stage.^42^

The accumulation of PI3P‐binding domain proteins enhances ATG binding, promoting membrane extension. Additionally, two ubiquitin‐like protein modification systems are essential here. In one system, ATG7 and ATG10 mediate the conjugation of ATG12 to ATG5, which then binds to ATG16 to form the ATG12–ATG5–ATG16 complex.^43^, ^44^ In the second system, ATG8 (LC3) is first cleaved by ATG4 into LC3‐I, activated by ATG7, and then conjugated with phosphatidylethanolamine (PE) by ATG3 to form LC3–PE (LC3‐II).^45^ The Atg12–Atg5 conjugate, acting as an E3 ligase, facilitates this final step, aiding membrane elongation. Despite the well‐characterized mechanism of the Atg8/LC3 coupling system, its precise function in autophagy remains to be fully elucidated.

Upon expansion and closure, the autophagosome matures and subsequently fuses with a lysosome to form an autolysosome, completing the autophagic process.^46^ Recent findings indicate that the fusion of autophagosomes and lysosomes involves various soluble NSF‐attachment protSNARE protein pairs, including Q‐SNAREs on autophagosomes (e.g., STX17 and SNAP29) and R‐SNAREs on lysosomes (e.g., VAMP8 or VAMP7).^47^ During this fusion process, LC3 proteins associated with autophagosomes are degraded and subsequently recycled, with degradation by‐products such as amino acids and fatty acids reintroduced into the cytoplasm for cellular reuse.

Unlike other forms of PCD, autophagy is predominantly regarded as a cell‐protective mechanism. However, its role in PCD is complex, as excessive autophagy can have harmful effects. This dual nature is particularly evident in cancer contexts.^48^ On one side, autophagy acts as a tumor suppressor by removing defective organelles, reducing oxidative stress, and preventing DNA damage.^49^ Conversely, in advanced tumor stages, autophagy promotes tumor progression by meeting the energy demands of tumor cells under the pressures of the tumor microenvironment and mitigating cytotoxicity.^50^ Given the dynamic and intricate nature of autophagy, while targeting autophagy‐related pathways holds therapeutic promise, comprehensive fundamental research is essential to fully elucidate the molecular mechanisms involved.

### 2.3 Necroptosis

Necrosis has historically been viewed as an unregulated process, while necroptosis, a recently identified mode of PCD, incorporates features of both necrosis and apoptosis.^51^ Although necroptosis shares genetic characteristics with necrosis, its molecular pathways are markedly distinct. Initially defined as the inhibition of caspase‐independent necrosis through treatment with necrostatin‐1 (nec‐1), necroptosis is characterized by its dependence on specific signaling mechanisms.^52^ Both necroptosis and extrinsic apoptosis utilize a shared extracellular receptor complex; however, most normal cells default to apoptosis due to their lower propensity for inducing inflammation. In circumstances where external factors—such as genetic alterations, molecular interactions, or pharmacological interventions—fail to elicit extrinsic apoptosis, the prodeath signals intended to activate apoptosis can instead trigger necroptosis, a highly inflammatory form of cellular demise.^53^

The induction of necroptosis is initiated by death receptors, including TNFR1, DR4/DR5, and FAS, localized on the cell membrane, as well as pattern‐recognition receptors.^54^ The downstream signaling pathway is regulated by three critical molecules: MLKL (mixed lineage kinase domain‐like pseudokinase), RIPK1 (receptor‐interacting serine/threonine kinase 1), and RIPK3. The occurrence of necroptosis is contingent upon subsequent modifications within complex I.^55^ A pivotal step in initiating extrinsic apoptosis is the formation of a secondary complex involving RIPK1 and activated caspase‐8, which also functions as a critical inhibitor of necroptosis. In contrast to apoptosis, inhibition of caspase‐8 leads to the accumulation of unprocessed RIPK1 within complex II, resulting in automatic cross‐phosphorylation between RIPK1 and RIPK3.^56^ The protein complexes formed during this process, referred to as necrosomes and ripoptosomes, represent dysregulated reorientations of complex I that mediate necroptosis and apoptosis. The phosphorylation of RIPK3 subsequently triggers the recruitment and phosphorylation of MLKL, facilitating its translocation to the plasma membrane, where it forms pores that compromise membrane integrity.^57^ This disruption results in an influx of water and Na^+^, alongside an efflux of K^+^, culminating in cellular swelling, disrupted membrane potential, and ultimately cell lysis.^58^

Given the substantial interrelationship between necroptosis and apoptosis, these modes of cell death are rarely observed in isolation. In 2019, Malireddi et al.^59^ introduced the concept of PANoptosis, a form of total cell death characterized by the simultaneous occurrence of pyroptosis, apoptosis, and necroptosis, which cannot be adequately explained by any single process. Subsequent investigations into PANoptosis have highlighted the key, coordinated role of caspase‐8, which not only inhibits RIPK1‐mediated necroptosis but also participates in the formation of pyroptotic inflammasomes and the release of inflammatory cytokines.^60^ The cellular stress responses of autophagy and necroptosis, though diametrically opposed, also exhibit notable interactions.^61^ Notably, RIPK3 has been shown to interact with and phosphorylate AMPK, leading to its activation, which in turn regulates ULK1 and BECN‐1. However, TNF‐induced necroptosis can impede the lysosomal degradation of autophagosomes, likely due to dysregulation of the SNARE complex.

### 2.4 Pyroptosis

The process of pyroptosis, akin to necroptosis, involves the formation of specialized pores in the cell membrane, culminating in a regulated form of cell death.^62^ Activation of the inflammasome and caspase activity is crucial in pyroptosis, which is inherently inflammatory.^63^ Various studies have shown that human caspase‐1, ‐4, and ‐5, as well as mouse caspase‐1 and ‐11, can induce pyroptosis by cleaving Gasdermin D (GSDMD). The cleaved fragments of GSDMD then oligomerize with their N‐terminal counterparts, resulting in pore formation in the plasma membrane.^64^ Consequently, GSDMD pores represent the primary mechanism underlying pyroptosis. Activated caspases also cleave and activate several precursors of inflammatory cytokines, including pro‐IL‐1β and pro‐IL‐18, allowing the mature forms of IL‐1β and IL‐18 to be released extracellularly through the GSDMD pores via the flow of intracellular fluid and ions.^65^

The inflammasome serves as a platform for caspase‐1 activation, with its assembly induced by damage‐associated molecular patterns (DAMPs) and pathogen‐associated molecular patterns (PAMPs). This complex consists of various inflammatory sensors that aggregate to form distinct types, including the NLRP3, AIM2, NLRP1, PYRIN, and NLRC4 inflammasomes.^66^ Certain sensors, such as AIM2, directly bind to specific DAMPs or PAMPs, with AIM2 recognizing viral or bacterial double‐stranded DNA and NLRC4 binding to bacterial flagellin and related proteins.^67^, ^68^ Other sensors, such as NLRP3 and PYRIN, detect changes induced by DAMPs and PAMPs, with NLRP3 sensing ATP, uric acid crystals associated with gout, and various toxins.^69^, ^70^, ^71^ The NLRP1 inflammasome is significantly activated by anthrax lethal toxin, Toxoplasma gondii, and muramyl dipeptide.^72^ Thus, pyroptosis occupies a unique role in host defense due to its distinctive mechanism. In contrast to classical pyroptosis mediated by caspase‐1, caspase‐4 and ‐5 (analogous to caspase‐11 in mice) trigger pyroptosis via a noninflammasome pathway that directly recognizes bacterial lipopolysaccharides, initiating pyroptosis more directly.^73^

Although GSDMD is pivotal in pyroptosis, other members of the gasdermin family also warrant attention.^74^ In apoptosis, caspase‐8, activated by death receptors, serves as the initiator and facilitates the activation of caspase‐3. Research indicates that GSDME can mediate the conversion of caspase‐3‐mediated apoptosis into pyroptosis, with its N‐terminal fragment inducing pore formation in the cell membrane.^75^ Additionally, while the precise function of GSDMC remains unclear, it has been confirmed that α‐ketoglutarate can trigger pyroptosis through caspase‐8‐mediated cleavage of GSDMC.^76^ This potential nonclassical pyroptosis pathway may represent a promising avenue for future research.

### 2.5 Ferroptosis

Ferroptosis, a newly identified form of cell death proposed by Dixon, is characterized by an imbalance of iron‐related lipid peroxides within the cell.^77^ The precise mechanism by which lipid peroxides compromise plasma membrane integrity remains unclear. Ferroptosis exhibits distinct morphological and genetic features compared with apoptosis, pyroptosis, and autophagy.^78^ Key factors driving this process include the generation of reactive oxygen species (ROS), the presence of phospholipids containing polyunsaturated fatty acid chains (PUFA‐PL), and increased iron accumulation.^79^

The manifestation of ferroptosis results from a delicate balance of intracellular redox reactions.^80^ Iron ions play extensive roles in key physiological reactions within the human body, making iron metabolic pathways particularly classical. Fe^3+^ is transported into the cell via the transferrin receptor located on the cell membrane, where it is subsequently reduced to Fe^2+^ by STEAP3. Fe^2+^ preferentially forms various iron‐binding complexes, while excess Fe^2+^ accumulates within cells, leading to unstable pools of iron. This accumulation can trigger ROS production through participation in the Fenton reaction.^81^ Ultimately, ferroptosis is driven by lipid peroxidation, with PUFAs being highly susceptible to this process and serving as primary targets. Multiple PUFAs may be involved, with acyl‐coenzyme A synthetase long‐chain family member 4 (ACSL4) playing a pivotal role by linking free PUFAs to coenzyme A, producing PUFA‐CoAs. These compounds then bind to phospholipids (PUFA‐PL), which are prone to oxidation by lipoxygenase, ultimately culminating in ferroptosis.^82^

Intricate antioxidant mechanisms within the cell typically prevent ferroptosis, even under conditions of iron overload or PUFA accumulation.^83^ Glutathione peroxidase 4 (GPX4) serves as the primary enzyme for the clearance of lipid peroxides within the cell. The uptake of cystine, a precursor for glutathione synthesis (GSH), occurs via the cystine/glutamate reverse transport system (System Xc^−^). GPX4 utilizes GSH as a substrate to exert its antioxidant effects.^84^, ^85^ Established inducers of ferroptosis include erastin and RSL3; erastin specifically targets System Xc^−^, inhibiting cystine uptake and leading to intracellular depletion of GSH.^86^ The reduction of GSH suppresses GPX4 activity, facilitating the accumulation of lipid peroxides.^87^ In addition to the classical GPX4‐centered regulatory pathways, several GPX4‐independent mechanisms for lipid peroxide clearance have been identified. The ferroptosis suppressor protein 1 (FSP1)–CoQ10–NAD(P)H pathway acts in parallel with GPX4 and GSH, collaboratively inhibiting lipid peroxidation and ferroptosis.^88^ FSP1 utilizes intracellular NADH to convert CoQ10 into its reduced form.^89^ Similarly, dihydroorotate dehydrogenase functions analogously to FSP1.^90^ Moreover, GCH1 produces the metabolite BH4, which removes lipid peroxides and aids in CoQ10 synthesis.^91^ As research advances, the understanding of the mechanisms underlying ferroptosis continues to expand beyond these pathways.

While ferroptosis remains significant, cuproptosis has emerged as a new area of interest.^92^ The discovery of ferroptosis has prompted investigations into metal‐dependent PCD. Excessive copper ions disrupt mitochondrial metabolic function by promoting the abnormal oligomerization of copper‐dependent fatty acylated proteins in the tricarboxylic acid cycle and reducing the levels of Fe–S cluster proteins. This phenomenon represents a distinct mode of PCD termed cuproptosis.^93^ Although the mechanisms of ferroptosis and cuproptosis differ considerably, evidence suggests a correlation between the two. Both iron and copper possess strong redox potentials, and copper can similarly induce ROS production via the Fenton reaction.^94^ The interaction between GSH and copper is complex, occurring concurrently; during physiological processes, excess intracellular copper ions can be sequestered, with GSH playing a critical role in this storage and potentially influencing copper metabolism and transport.^95^

## 3 BIOLOGICAL FUNCTIONS OF PCD

**FIGURE 2:**
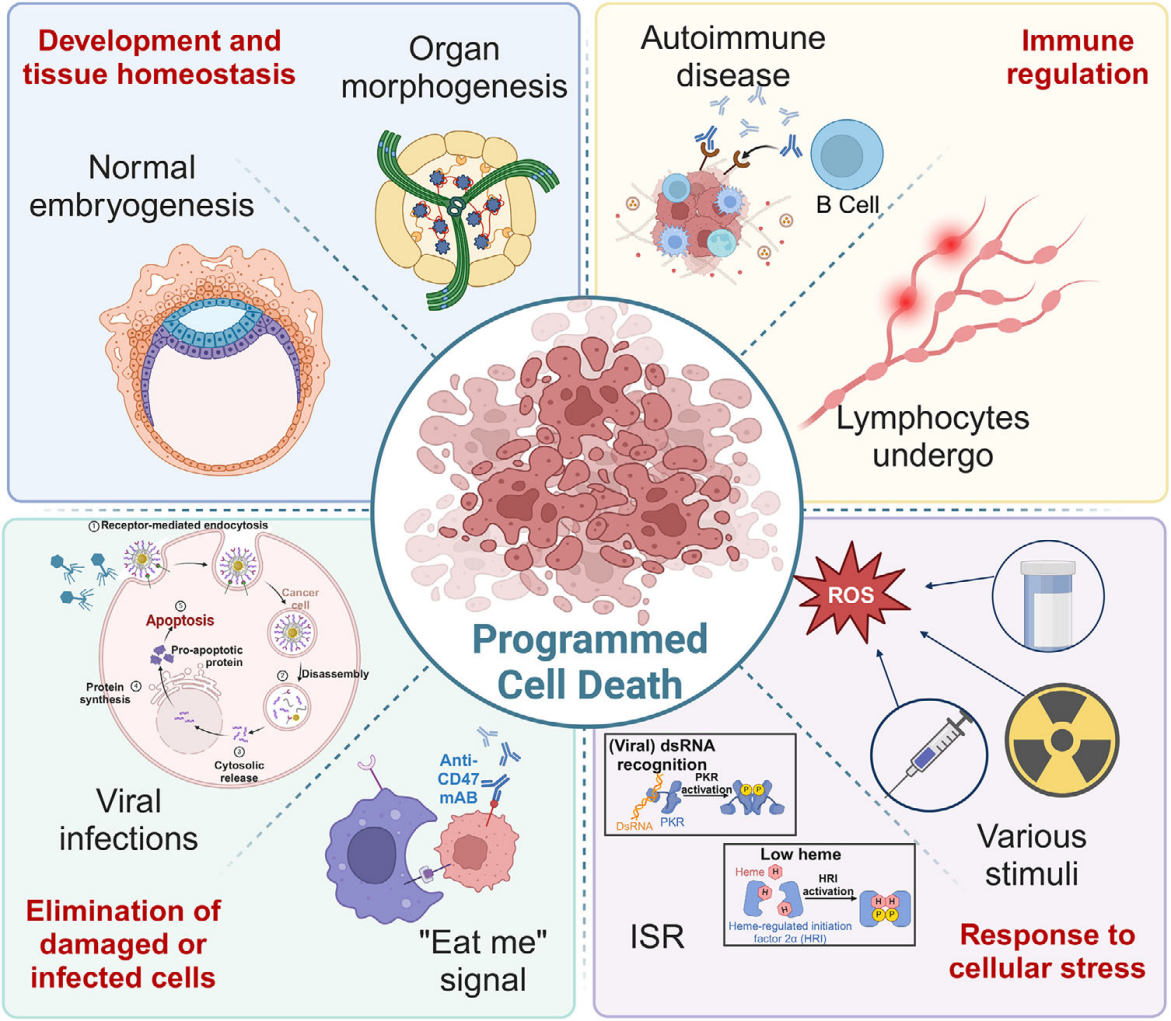
Biological functions of programmed cell death. Programmed cell death plays a vital role in development and tissue homeostasis, being closely linked to embryogenesis and organ morphogenesis. It is instrumental in immune regulation, serving as a key mechanism for the regulation of immune cells and the establishment and maintenance of lymphocyte pools. In addition to being an initial trigger for programmed cell death activation, it can also be induced by cellular stress. Moreover, PCD is essential for the clearance of damaged cells and the body's response to bacterial and viral infections. Figures were created with biorender.com.

Accurate PCD plays a critical role in biological processes throughout the entire lifespan of an organism. During early development, PCD contributes to organ and tissue formation by eliminating excess or unnecessary cells, ensuring the proper establishment and maintenance of complex structures. In the context of immune regulation, PCD fine‐tunes the immune response by facilitating the removal of activated immune cells after an infection has resolved, thus preventing excessive immune reactions and maintaining homeostasis. Furthermore, when cells encounter various forms of stress, such as DNA damage or oxidative stress, PCD is activated to eliminate irreparably damaged cells, thereby preventing the accumulation of potentially harmful components. This process is vital for eliminating damaged or infected cells, curtailing the spread of infections, reducing the risk of tumor formation, and safeguarding the overall health and integrity of the organism. In summary, PCD is an essential mechanism that ensures proper physiological function and survival (Figure 2).

### 3.1 Development and tissue homeostasis

Throughout growth and development, particularly during embryogenesis and organ morphogenesis, there is a regulated occurrence of cell degeneration and death. This cell death follows a specific spatiotemporal pattern, underscoring the critical role of PCD in both normal embryogenesis and organ development.^96^ Even when an organism successfully completes embryogenesis and exhibits normal physiological functions, it may still experience accidental injuries. Regeneration serves as a vital mechanism for regrowth and repair, facilitating tissue homeostasis even after significant injury, with PCD contributing to this restorative process.^97^

Research utilizing the development of *C. elegans* has led to significant advancements in the understanding of PCD, establishing it as an exemplary model organism for research.^98^ During the embryogenesis of *C. elegans*, a total of 1090 somatic cells are generated, of which 131 undergo apoptosis at specific developmental stages.^99^ Loss of function mutations in egl‐1, ced‐3, and ced‐4 result in the survival of all 131 cells, while the absence of the cell death suppressor ced‐9 leads to extensive cell death and embryonic lethality.^100^ The biological functions of PCD during embryogenesis primarily manifest in three key aspects: regulating cell quality, sculpting tissues, and eliminating transient structures or organs.

Embryogenesis entails the timely removal of unwanted and potentially harmful cells through PCD, which regulates cell quality and protects against viral infections, DNA damage, and cell cycle disturbances. For instance, aneuploidy is recognized as a factor contributing to suboptimal embryo quality; however, the incidence of aneuploidy significantly decreases as gestation progresses. In a mouse model of chromosomal mosaicism, the combination of autophagy and apoptosis has been validated as an effective mechanism for eliminating defective cells, ensuring that only chromosomally viable cells continue to advance during embryogenesis.^101^ The poor quality of embryonic cells may also stem from viral infections. Parvovirus B19 infection is associated with fetal edema and can stimulate erythrocyte mitosis, with autophagy expression upregulated in B19‐infected cells.^102^ Research indicates that the human placenta can protect extra‐placental cells from viral infections through autophagic processes.^103^ Once embryonic development is assured, PCD intricately sculpts tissue and organ structures by selectively eliminating specific cells. A classic example of this is the precise formation of fingers and toes, where apoptosis is the primary mechanism for eliminating the interdigital webbing.^104^ This apoptotic process occurs in the mesenchymal cells of the hands and feet, with the initiation site of PCD determining both finger and toe morphology, as well as the separation between metacarpals and phalanges. The formation of the atretic vaginal canal exemplifies how aberrant apoptosis can lead to organ morphogenesis defaults. Consequently, PCD plays a vital role in transforming solid tissues into hollow‐structured organs. During cardiac development, apoptosis is essential for the morphogenesis of the atrioventricular septum, valves, and vascular structures, with dysregulated levels of apoptosis linked to congenital heart diseases.^105^ Additionally, the elimination of transient structures or organs represents a critical function of PCD, as certain structures formed during embryogenesis are temporary and serve short‐term purposes. In female mammals, the Müllerian duct develops into the oviducts and uterus while regressing in males. Conversely, the Wolffian duct, which develops into the vas deferens, epididymis, and seminal vesicle in males, undergoes degradation in females.^106^ Apoptotic cells have also been identified in various organs, such as the gut and skin, during Xenopus laevis development, and cell death occurs in transitional organs like gills.^107^ This observation highlights an ongoing process of active tissue degeneration and remodeling within the organism.

In contrast to active tissue remodeling, the passive involvement of PCD in tissue regeneration primarily manifests through its protective capacity following severe injury. This regenerative process is exemplified by the hydra, which can regenerate a new head after experiencing head loss.^108^ In the context of tissue regeneration, apoptosis also plays a critical role in regulating cell number and proliferation during tissue remodeling, acting as a stimulating signal for the regenerative process.^97^ Bergmann and Steller.^109^ described this phenomenon as “apoptosis‐induced compensatory proliferation.” The apoptosis of cells resulting from stress or injury can trigger the proliferation of adjacent cells as a compensatory mechanism to maintain cellular homeostasis. However, if apoptotic cells that promote proliferation are not eliminated in a timely manner, it can lead to excessive tissue growth. Studies have shown that cells lacking caspase‐3 are unable to facilitate the proliferation of neighboring cells following radiation exposure, indicating the direct involvement of caspase‐3 in apoptosis‐induced proliferation. This finding may help address the issue of accelerated tumor growth during the early stages of radiation and chemotherapy.^110^ The phenomenon of apoptosis‐induced compensatory proliferation is not solely dependent on caspase‐3; it involves multiple signaling pathways. For example, JNK signaling plays a pivotal role in compensatory proliferation and wound healing in *Drosophila*.^111^ Additionally, research has demonstrated the collaborative action of the p53 and Dronc signaling networks in promoting compensatory proliferation and facilitating blastocyst formation.^112^ The significant clinical potential of apoptosis lies in its capacity to enhance tissue regeneration. Apoptotic vesicles derived from human deciduous pulp stem cells can influence pulp regeneration processes and activate autophagy in recipient cells, thereby inducing host angiogenesis in hypoxic‐ischemic environments.^113^ Such groundbreaking research underscores the irreplaceable role of PCD in development and tissue homeostasis.

### 3.2 Immune regulation

The establishment of the immune system is heavily dependent on the robust support provided by immune cells, which act as the effectors of the immune response. PCD, a highly regulated process that can be promptly initiated or reversed, serves as a pivotal mechanism for regulating immune cell populations.^114^ The presence of PCD is essential for establishing and maintaining lymphocyte pools, ensuring a consistent size throughout an individual's lifespan. The significance of PCD in immune regulation primarily manifests in the selection of immune cells and the modulation of immune responses.^115^ Dysregulation of PCD, whether excessive or insufficient, can lead to immune deficiencies or autoimmune diseases.

During development, lymphocytes undergo a stringent selection process that results in the elimination of the majority of lymphocytes. To ensure early survival, T cells and B cells must express functional T cell antigen receptors (TCRs) or B cell antigen receptors (BCRs). Lymphocytes that lack functional TCRs or BCRs do not receive the necessary signals to inhibit apoptosis and are cleared through a process termed “death by neglect.”^116^ Lymphocytes that express TCRs or BCRs are still at risk of apoptosis due to high affinity for autoantigens; this process, known as negative selection, is essential for acquiring immune tolerance toward autoantigens.^117^ In contrast, the response of cells with functional receptors to their autoantigens is comparatively weak, allowing these cells to differentiate into mature lymphocytes. The activation of TCRs, unlike BCRs, requires the simultaneous recognition of an antigenic peptide and major histocompatibility complex (MHC) molecules, a phenomenon known as “MHC restriction.” Consequently, an additional positive selection step is necessary for T cell development.^118^ T cells with MHC Class II‐specific TCRs typically retain CD4 expression while losing CD8 expression, whereas T cells with MHC Class I‐specific TCRs express CD8 without CD4. Lymphocyte selection based on these criteria is predominantly mediated by apoptosis.^119^

The regulation of lymphocyte development extends beyond the aforementioned selection processes and is influenced by various additional factors. The survival of developing lymphocytes relies on signals from cytokine receptors, with cytokines such as IL‐2, IL‐3, IL‐4, IL‐7, IL‐9, and IL‐15 playing critical roles in promoting their development as essential survival factors for the immune system.^120^ The common γ chain (γc) shared by these cytokine receptors is considered pivotal; mice deficient in the γc‐associated kinase Jak3 exhibit severe combined immune deficiency.^121^ These cytokines enhance cell survival by upregulating antiapoptotic genes. For example, enforced expression of the Bcl‐2 gene in T cells from IL‐7R alpha‐deficient mice significantly restores thymic positive selection, T cell counts, and overall function.^122^

When a pathogen triggers an immune response, the rapid expansion of antigen‐specific lymphocytes becomes essential. Consequently, the establishment of checkpoints is essential for ensuring the selective survival and proliferation of lymphocytes upon antigen encounter. Modulation of costimulation is a key mechanism in this process; in the absence of adequate costimulation, lymphocytes fail to achieve full activation and undergo apoptosis at an increased rate.^123^ T cells primarily rely on CD28, whereas B cells require CD19 and CD40 for effective costimulation. Costimulatory signals induce the expression of cytokines and antiapoptotic molecules, providing protective effects against cell death.^124^, ^125^ PCD plays a regulatory role for activated lymphocytes, restoring cellular homeostasis during the regression phase of immune responses. Studies have shown that mouse genetic models deficient in CD95 (Fas) and CD178 (FasL), as well as individuals with autoimmune lymphoproliferative syndrome, exhibit lymphoproliferative disorders.^126^ Thus, the TNF family of receptors is crucial during immune regression.^127^ Additionally, Fas serves as a classical initiation signal for extrinsic apoptosis, further substantiating the fundamental role of apoptosis in regulating immune responses.

### 3.3 Response to cellular stress

The interplay between PCD and cellular stress is bidirectional, warranting a closer examination of their relationship. On one hand, stress serves as a prototypical extracellular stimulus, acting as an initial trigger for the activation of various forms of PCD, including apoptosis, necroptosis, and pyroptosis, in response to physical, chemical, and mechanical stimuli. On the other hand, stress can also function as a critical factor that induces cell death following the initiation of PCD pathways, such as ROS stress and toxic protein stress during ferroptosis and cuproptosis, respectively. Thus, the delicate balance between PCD and cellular stress ultimately determines cellular fate. This discussion primarily focuses on how PCD responds to cellular stress.

Stress often poses a significant threat to cell survival. When the detrimental stimulation exceeds the cells’ capacity to cope, it invariably triggers PCD, as severely damaged cells are destined for deactivation or elimination. Previous studies have demonstrated that osmotic stress, characterized by a lower osmotic pressure in intracellular fluid compared with extracellular fluid, leads to intracellular water loss, DNA breakage, and disruption of normal cellular signaling.^128^ The molecular mechanisms underlying cell death induced by osmotic stress have been elucidated, revealing a pivotal role in RIPK3 activation‐mediated necroptosis.^129^ Similar findings have been validated in the context of heat stress, where heat‐induced upregulation of ZBP1 expression activates RIPK3.^130^ Consequently, elevated temperatures not only inflict physical damage but also trigger necroptosis. Previously, it was believed that heat injury exclusively triggered the heat shock response (HSR); however, it is now understood that various stress factors can elicit this response. Heat shock proteins (HSPs), highly conserved throughout evolution, play a critical role in maintaining physiological stability. Facilitating the synthesis and release of HSPs contributes to cellular homeostasis.^131^ Overexpression of HSP90 generally promotes cellular protection, while its inhibition or knockdown can decrease cell viability and mediate PCD. For instance, knockout of HSP90 has been shown to effectively trigger apoptosis in ovarian cancer cells, presenting a promising therapeutic strategy for addressing multidrug‐resistant forms of the disease.^132^ The disruption of the delicate balance between oxidative and antioxidant mechanisms is a common form of cellular stress, with the identification of ferroptosis underscoring the pivotal role of ROS in determining cell fate. Additionally, oxidative stress is believed to mediate apoptosis.^133^ For example, oxidative stress and subsequent neuronal apoptosis following traumatic brain injury significantly contribute to resultant damage. Research indicates that upregulation of Uqcr11 can mitigate neuronal apoptosis and facilitate recovery of brain function by inhibiting oxidative stress.^134^ Similarly, oxidative stress has been implicated in the damage of nucleus pulposus cells, leading to intervertebral disc degeneration.^135^ Thus, PCD can thus be regarded as a stress response mechanism aimed at preserving homeostasis.

The ability of living organisms to adapt and evolve over extended periods, often referred to as the survival of the fittest, hinges on their capacity to sense environmental stress and initiate complex physiological mechanisms for adaptive responses. Successful adaptation ensures survival, whereas failure in this regard can lead to PCD, as previously discussed.^136^ A classic example of PCD contributing to cellular adaptation and survival is the efficient clearance of apoptotic cells by macrophages in tissues, accompanied by the transmission of potent anti‐inflammatory signals.^137^ The mechanism of autophagy has been elucidated, revealing that nutrient deprivation triggers autophagy in most cells within an organism, enabling them to dismantle their own components as a survival strategy. However, cells possess additional adaptive mechanisms. Eukaryotes exhibit a conserved adaptive signaling pathway known as the integrated stress response (ISR) in reaction to cellular stress.^138^ When pressure sensor molecules activate signaling networks within cells, specialized mechanisms respond to failures in maintaining or re‐establishing proteostasis. The unfolded protein response (UPR) leads to the aggregation of misfolded proteins in the ER, while the HSR occurs in the cytoplasm, enhancing the cell's ability to fold and degrade proteins.^139^ The ISR is capable of addressing both endoplasmic and cytoplasmic stress conditions, distinguishing it from other stress responses. Notably, the ISR can be activated alongside UPR and HSR, further amplifying its effectiveness. By reprogramming cellular translation mechanisms, the ISR plays a critical role in promoting the survival of stressed cells.^140^ It attenuates the overall rate of translation initiation while enhancing the translation of specific messenger RNAs (mRNAs). These dual processes reprogram gene expression to maintain cellular homeostasis, uphold protein folding capacity, facilitate differentiation, and respond to injurious stimuli. When stressors cannot be alleviated, the ISR ultimately triggers apoptosis as a means to eliminate damaged cells.

### 3.4 Elimination of damaged or infected cells

The clearance of damaged cells from the human body is a critical process that merits detailed discussion, particularly in the context of how cells mount effective responses to eliminate infected cells and limit pathogen replication during bacterial and viral infections. Findings indicate that PCD plays a significant role in this mechanism.

As noted, apoptosis is primarily activated when the extent of cellular damage exceeds manageable limits. Given that billions of cells sustain damage and subsequently undergo cell death daily within the human body, it is critical to eliminate these cells effectively. Efferocytosis, a multistage mechanism for clearing apoptotic cells, is mediated by phagocytes and serves to prevent the release of potentially harmful cellular contents that could trigger an autoimmune response^141^ The principle of efferocytosis relies on the presence of specific “eat me” signals on the surface of apoptotic cells, which are recognized by surrounding phagocytes, such as macrophages and dendritic cells.^142^ The most common “eat me” signal is phosphatidylserine, which is typically localized on the inner side of the cell membrane in healthy cells but becomes exposed on the outer surface upon cellular damage. Once recognized, phagocytes bind tightly to apoptotic cells via their receptors and subsequently engulf them by extending their plasma membrane to form an intracellular structure known as a phagosome. This phagosome then fuses with a lysosome, creating a digestive compartment within the phagocyte.^143^ The subsequent breakdown and digestion of the engulfed cell, followed by the recycling or excretion of its residues, is essential for the stable clearance of damaged cells, akin to the processes observed in autophagy. Research into “eat me” signals as self‐clearing markers for damaged cells has consistently been a focal point in studies of PCD. The Ca^2+^‐activated phospholipid scramblase TMEM16F and the caspase‐dependent phospholipid scramblase Xkr8 have been identified as key factors in lipid disorder during this process.^144^ Recently, the mechanism by which protein Xkr4 mediates the expression of the “eat me” signal has also been elucidated. Severe cellular damage triggers caspase‐mediated cleavage of XRCC4, releasing its C‐terminal fragment from the nucleus into the cytoplasm. This fragment subsequently interacts directly with the Xkr4 dimer, activating and regulating it, which in turn stimulates the rearrangement of the phospholipid bilayer on the plasma membrane. Consequently, ectopic expression of phospholipid molecules occurs, further facilitating the recognition and clearance of damaged cells.^145^

Bacterial reproduction occurs through a straightforward process known as binary fission, whereas viruses rely entirely on the intracellular environment of host cells, necessitating their inhabitation for replication. Immediate cell death following infection can impede the transmission pathway, and apoptosis provides a plausible mechanism for eliminating infected cells. Numerous bacteria and viruses possess apoptosis inhibitors that play pivotal roles in their virulence, particularly among viruses.^146^ Apoptosis is a key component of the immune response triggered by infection, which can be initiated via extrinsic and intrinsic pathways as well as by cytotoxic T lymphocytes (CTLs) or natural killer cells. These immune cells release granzyme and activate apoptotic caspases.^147^ Granzyme B, a member of this group, facilitates MOMP and subsequent activation of caspase‐9 by cleaving BID, a BH3‐only protein.^148^ Studies indicate that CTLs can eliminate an average of 2 to 16 virus‐infected cells daily.^149^ FasL can also be expressed on the surface of CTLs, triggering extrinsic apoptosis. Therefore, the targeting of immune cells induced by infection often exacerbates disease development, as apoptosis aimed at eliminating infected cells is activated. The pathogenesis of HIV infection primarily involves the impairment of CD4+ T cells, with increasing evidence supporting the association between HIV‐induced apoptosis and the upregulation of death receptors by various HIV proteins.^150^ This suggests that the process of viral infection promoting apoptosis may be mediated not only by the immune system but also through the indirect activation of apoptosis genes by viral proteins. For example, human parvovirus B19 can induce apoptosis in red blood cells by recruiting Bax and Bad proteins, thereby upregulating the activity of caspase‐3 and caspase‐9.^151^ However, certain viruses have evolved various mechanisms to ensure their prolonged survival within the host by interfering with apoptosis, effectively evading clearance of infected cells and facilitating long‐term incubation.^152^ The inhibition of death receptor signaling represents a common pathway. For instance, respiratory syncytial virus enhances the expression of the antiapoptotic gene IEX‐1L, effectively suppressing apoptosis induced by TNF‐α.^153^ When a virus requires long‐term survival within a host cell, it synthesizes antiapoptotic proteins to counteract potential threats posed by apoptosis. For example, the E6 protein of human papillomavirus (HPV) disrupts apoptosis signaling at the p53 level, while the ribonucleotide reductase ICP10 of herpes simplex virus directly interacts with caspase‐8.^154^, ^155^, ^156^ Similarly, the M36 protein encoded by murine cytomegalovirus inhibits extrinsic apoptosis mediated by caspase‐8.^157^ In summary, apoptosis plays a significant role in the elimination of infected cells, particularly during viral infections.

## 4 DISEASES ASSOCIATED WITH DYSREGULATED PCD

**FIGURE 3:**
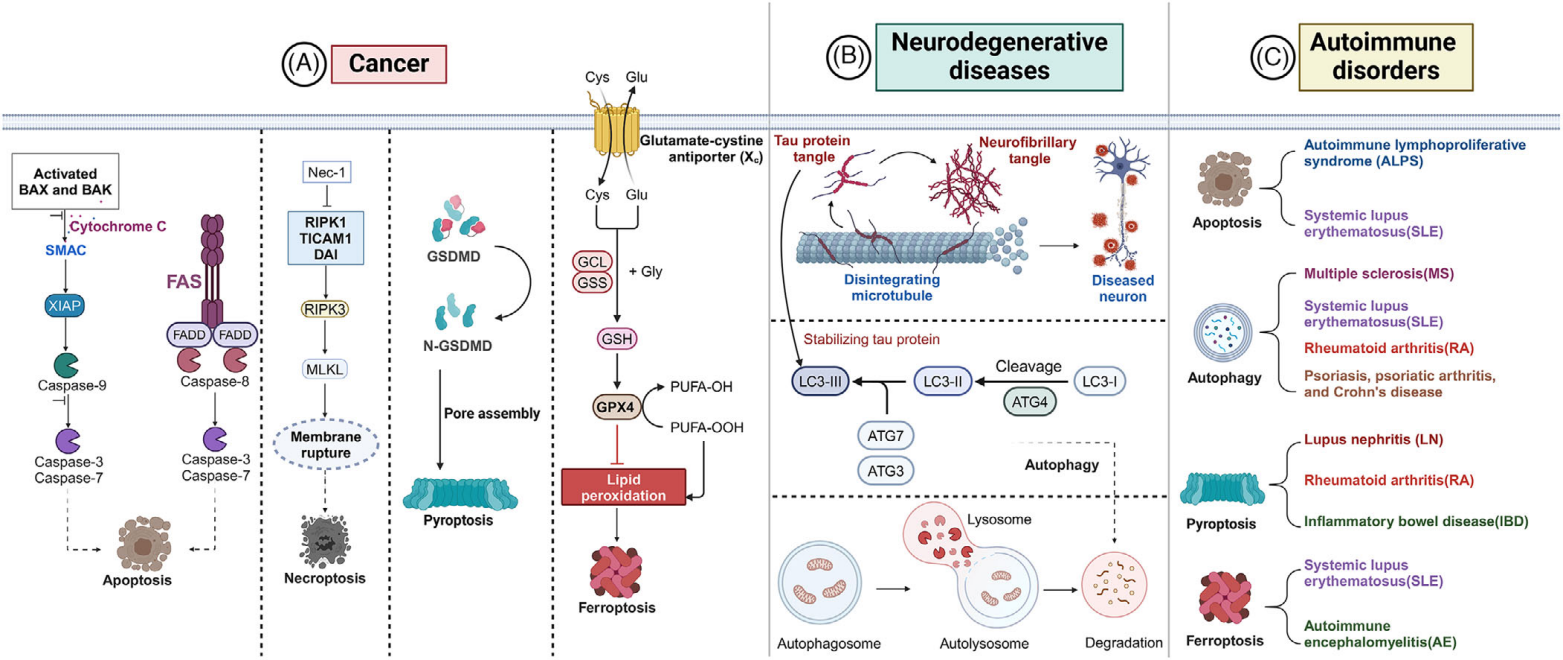
Diseases associated with dysregulated programmed cell death. This summary highlights various diseases associated with programmed cell death, including tumors, neurodegenerative diseases, and autoimmune disorders. (A) Programmed cell death related to tumors encompasses apoptosis, necroptosis, pyroptosis, and ferroptosis. (B) The dysregulation of programmed cell death in neurodegenerative diseases is primarily linked to dysfunctional autophagy. (C) In autoimmune diseases, programmed cell death predominantly involves apoptosis, autophagy, pyroptosis, and ferroptosis. TICAM1, Toll‐like receptor adaptor molecule 1; DAI, DNA‐dependent activator of IRFs. Figures were created with biorender.com.

Dysregulated PCD is closely linked to numerous diseases, posing significant challenges in the medical field. Cancer, neurodegenerative diseases, and autoimmune disorders are currently subjects of extensive research. In cancer, the hallmark characteristic is the infinite proliferation of cells, with the evasion of apoptosis serving as a critical component of tumorigenesis. This evasion facilitates tumor growth and metastasis, complicating cancer treatment. Conversely, when autophagy is impaired, abnormal protein aggregates accumulate within neurons, leading to cellular dysfunction and eventual cell death, thereby contributing to the progression of neurodegenerative diseases. Autoimmune disorders also exhibit dysregulated PCD, which represents an opposite dynamic compared with the immune regulation aspects of PCD. Ultimately, advancing therapeutic strategies and improving patient outcomes remain the primary goals of investigating PCD (Figure 3).

### 4.1 Cancers

The pathological state of cancer is characterized by uncontrolled cell proliferation, with the fundamental principle of cancer treatment centered on halting this excessive growth and effectively eradicating these “immortalized” cells. PCD provides a potent avenue for actively manipulating cellular fate, making the discovery of novel forms of PCD particularly promising for advancing cancer research. Apoptosis serves as a key regulatory mechanism within the cell cycle, enabling the timely removal of nonfunctional, harmful, abnormal, and misplaced cells. Given that the mechanisms of apoptosis are the most thoroughly studied, and multiple strategies exist to target its key proteins, apoptosis has long been regarded as an essential mechanism for preventing tumor emergence. One defining characteristic of tumor cells is their evasion of apoptosis, with reduced apoptosis or resistance to it playing a significant role in tumorigenesis. Generally, apoptosis evasion during tumorigenesis involves three primary mechanisms: disruption of the balance between proapoptotic and antiapoptotic proteins, inhibition of caspase activity, and impairment of death receptor signaling pathways.^158^

A diverse range of proapoptotic and antiapoptotic proteins plays pivotal roles in the molecular mechanisms of apoptosis, with the ratio of these proteins—rather than their absolute quantities—ultimately dictating the fate of apoptosis. Among these proteins, the Bcl‐2 family is a quintessential example. Given the extensive size of the Bcl‐2 family, dysregulated apoptosis can result from the overexpression of one or more antiapoptotic proteins, one or more proapoptotic proteins, or a combination of both.^12^ Aberrant overexpression of prosurvival Bcl‐2 proteins has been linked to the pathogenesis and poor prognosis of various malignancies. For instance, overexpression of BCL‐2, myeloid cell leukemia 1 (MCL‐1), and BCL‐XL has been observed in lymphomas and leukemias.^159^, ^160^ Elevated levels of BCL‐XL have also been noted in several cases of colorectal cancer, suggesting that BCL‐XL inhibition may enhance the efficacy of chemotherapy for this disease.^161^ Conversely, reduced levels of proapoptotic Bcl‐2 family proteins, such as BIM and NOXA, have been documented in various human cancers.^162^, ^163^ BAX deficiency has been identified in several cancers, including endometrial and colon cancers.^164^, ^165^ Additionally, the involvement of Bcl‐2 family members in resistance to tumor therapies highlights their potential efficacy in overcoming chemotherapy and immunotherapy resistance through their role in apoptosis.^166^ Dysregulation of endogenous caspase inhibitors, known as IAPs, has been widely reported in numerous cancer types. In the context of ovarian cancer with BRCA1 mutations, administration of IAP inhibitors has demonstrated significant antitumor effects in mouse models.^167^ This indicates that inhibiting caspase activity—the key mediator of apoptosis—through alternative approaches can effectively promote evasion of apoptosis, irrespective of initiating or executing caspases. Downregulation of caspase‐9 has been associated with poor prognosis in colorectal cancer.^168^ Furthermore, reduced activity of caspase‐3 may contribute to chemotherapy resistance in breast cancer.^169^ Notably, multiple caspases can be downregulated within the same tumor, and further investigation is warranted regarding the contribution of decreased caspase activity to reduced pyroptosis levels and subsequent tumor formation, given the integral role of caspases in pyroptosis.

Interestingly, in tumors such as glioblastoma, caspase‐8 expression is preferentially preserved; elevated levels of caspase‐8 may indicate a poorer prognosis, suggesting that in this context, the protease loses its apoptotic activity while acquiring additional functions.^170^ Extrinsic apoptosis also warrants attention, as impaired death receptor function or decreased levels of death signals contribute to tumor development. For instance, the expression of FasL may serve as a potential biomarker for the prognosis of nasopharyngeal carcinoma.^171^ The absence of Fas and the dysregulation of FasL, DR4, DR5, and TRAIL may potentially contribute to the pathogenesis of cervical cancer.^172^ However, some tumor cells exhibit resistance to CD95L‐induced apoptosis, which complicates the role of the CD95–CD95L interaction. This phenomenon may promote tumor growth by fostering inflammation, regulating immune cell homeostasis, or sustaining tumor cell survival in a dormant state. Relevant studies have illustrated the dualistic nature of the death receptor pathway.^173^ Overall, while the significance of apoptosis evasion in tumorigenesis is well established, the emergence of conflicting findings underscores the complexity of the relationship between apoptosis and tumor biology, necessitating further research in this area.

The field of tumor therapies aimed at recovering apoptosis has progressed significantly and has been extensively studied. A detailed exploration of these therapies will be presented in Section 5.2, which focuses on targeted treatments for specific diseases. The discovery of other forms of PCD as modalities of cellular demise is expected to provide a foundation for the development of novel tumor therapies, and this concept has already seen successful implementation. For instance, the activation of necroptosis is mediated by the phosphorylation of RIPK1 and RIPK3, which in turn activates MLKL. Consequently, targeting RIPK1, RIPK3, and MLKL presents promising therapeutic strategies. Several small molecules targeting RIPK1 and RIPK3 have been identified, including some that have received United States Food and Drug Administration (US FDA) approval.^174^ For example, administration of chloroquine (CQ) significantly upregulates RIPK3, inducing apoptosis in colon cancer cells.^175^ Additionally, FTY720 has shown efficacy in lung cancer by activating PP2A‐RIPK1‐dependent necroptosis.^176^ However, RIPK1 and RIPK3 may not always serve as optimal therapeutic targets; under certain pathological conditions, RIPK1 may not be the primary mediator of necroptosis, and both RIPK1 and RIPK3 are involved in cellular inflammatory responses.^177^ The failure of multiple clinical trials investigating RIPK1 for various diseases further highlights this issue, suggesting that targeting MLKL may yield greater efficacy. The compound tanshinol A has been shown to elevate intracellular ROS and MLKL levels in lung cancer.^178^ Conversely, SKP2‐mediated degradation of MLKL contributes to cisplatin resistance in non‐small cell lung cancer (NSCLC) cells.^179^ The GSDM family associated with MLKL holds significant potential for further development, given the similarities between pyroptosis and necroptosis. Notably, metformin induces GSDMD‐mediated pyroptosis in nasopharyngeal carcinoma cells and triggers GSDME‐dependent caspase‐3 cleavage.^180^, ^181^ The potential toxicity of therapies based on necroptosis and pyroptosis, both of which are highly proinflammatory programmed cellular processes, requires careful consideration. Furthermore, the emerging concept of ferroptosis has gained traction in cancer research, presenting a novel avenue for exploration. Any factor that elevates intracellular ROS levels can induce ferroptosis. For instance, lupeol induces oxidative stress imbalance, significantly increasing ROS levels in nasopharyngeal carcinoma cells, indicating its potential for treatment.^182^ Among the various regulatory mechanisms of ferroptosis, GPX4 plays an indispensable role. Recent studies have identified several small molecules that effectively inhibit GPX4, including ML210, ML162, DMOCPTL, and FINO2.^183^ Additionally, System Xc^−^ is a prominent target for classic antioxidants, and restricting cystine input can effectively induce ferroptosis. Notably, several drugs, including US FDA‐approved sorafenib and sulfasalazine, have been identified as inhibitors of System Xc^−^, demonstrating remarkable antitumor effects.^184^ Overall, most ferroptosis‐related tumor therapies primarily target GPX4 and System Xc^−^,^185^ but additional pathways and molecules within the ferroptosis mechanism warrant further exploration. Although the complete mechanism underlying cuproptosis remains elusive, its potential as a tumor growth inhibitor is evident. Several prognostic models related to cuproptosis have been studied, particularly for head and neck squamous cell carcinoma.^186^ Furthermore, it is essential to consider the potential interrelationship between ferroptosis and cuproptosis, as shown in studies of primary liver cancer where ferroptosis activators such as sorafenib and erastin enhance the effects of cuproptosis by increasing copper‐dependent lipoylated protein aggregation and inhibiting the synthesis of the intracellular copper chelator GSH.^187^ Despite these advancements, the application of numerous targets still lacks comprehensive fundamental research and clinical translation, primarily due to inherent challenges in clinical application. It is imperative to conduct more detailed studies on the role of these targets within the human body to ensure they do not disrupt the normal progression of other biological pathways.

### 4.2 Neurodegenerative diseases

The process of autophagy, which involves the degradation and recycling of intracellular components, is widely acknowledged for its role in cellular waste management and recycling within the organism. In addition to various stimuli, such as nutritional deprivation and oxidative stress, protein aggregation significantly contributes to the activation of autophagy. During protein production, transportation, and storage, numerous factors can lead to protein aggregation. This aggregation is a critical characteristic of neurodegenerative diseases, and autophagy plays a vital role in degrading proteins that are prone to aggregation, thereby maintaining cellular homeostasis in nonstarving cells.^188^ However, postmitotic neurons cannot mitigate unwanted protein accumulation through cellular division. Consequently, dysregulated autophagy is associated with the pathogenesis of neurodegenerative disorders characterized by protein aggregation, including tauopathies, Alzheimer's disease (AD), and Parkinson's disease (PD)^189^

Tauopathies encompass a spectrum of neurological disorders characterized by the aggregation and formation of tau proteins into intracellular tangles, including progressive supranuclear palsy (PSP), corticobasal degeneration (CBD), and frontotemporal dementia (FTD).^190^ Studies indicate that dysautophagy manifests in various forms, all closely linked to tau protein aggregation. In patients with CBD and PSP, colocalization of Tau, LC3, and p62 has been observed, while patients with AD exhibit reduced p62 expression.^191^ Additionally, tau has the capacity to bind to lysosomal membranes, disrupting their permeability. Importantly, tau aggregation‐induced lysosomal dysfunction has been identified as one of the pathogenic mechanisms underlying FTD.^192^ Elevated levels of lysosomal components, including lysosomal‐associated membrane protein 1 (LAMP1) and cathepsin D, have also been found in patients with CBD and PSP.^193^ Dysregulated CMA, in addition to impaired macrophage function, further accelerates the accumulation of tau proteins. Pathogenic tau expression can specifically inhibit CMA in neurons.^194^ In mouse models, acetylated tau impairs CMA and facilitates the pathological propagation of tau, with similar molecular mechanisms observed in the brains of patients with tauopathies.^195^ In AD, intracellular tau tangles typically precede the formation of extracellular amyloid β (Aβ) plaques, and a complex interplay exists between Aβ and autophagy.^196^ Autophagy activation has been observed in microglia within AD mouse models, particularly in disease‐associated microglia localized around amyloid plaques.^197^ Moreover, faulty autolysosome acidification in the AD mouse model can lead to the accumulation of Aβ in neurons due to autophagic dysfunction.^198^ In PD, the primary role of α‐synuclein is related to synaptic function, with the accumulation of misfolded α‐syn and its aggregation into protein inclusion bodies known as Lewy bodies representing key steps in the genesis and progression of the disease. Normally, α‐synuclein is cleared through autophagy; however, its aggregates can directly disrupt the autophagy‐lysosomal pathway by decreasing ATG7 levels and increasing mTOR levels, leading to defects in autophagy initiation.^199^, ^200^ In both hereditary and sporadic forms of PD, α‐synuclein accumulation may stimulate autophagosome formation to meet increased degradation demands, yet excessive α‐synuclein expression can impede autophagosome biogenesis.^201^, ^202^ Aging significantly increases the risk of neurodegenerative diseases in humans, and a decline in autophagic processes has been observed with age. In most organs and tissues, CMA activity decreases as organisms age, primarily due to the reduced stability of LAMP2A on lysosomal membranes in older individuals.^203^ Consequently, the progressive decline in macrophage function associated with aging may result in the accumulation of toxic proteins within neurons, thereby impairing their overall health. Substantial evidence suggests that dysregulated autophagy is a key mechanism underlying aberrant protein aggregation and is extensively implicated in the pathogenesis and progression of neurodegenerative disorders.

Reducing protein levels presents an effective strategy for combating neurodegenerative diseases, which are primarily driven by protein misfolding and aggregation. Targeting autophagy emerges as a promising and efficacious therapeutic approach, to be discussed in detail in the subsequent Section (5.2.2). It is important to consider the involvement of other forms of PCD in treating neurodegeneration, as they can exert significant effects. The protective role of autophagy contrasts sharply with the detrimental impacts of oxidative stress and apoptosis, both of which pose substantial threats to neurons. Consequently, a primary objective in neurodegenerative treatment is to mitigate neuronal oxidative stress and apoptosis.^204^ Neurotrophic factors represent key pharmacological agents with antiapoptotic properties, specifically targeting neuronal apoptosis and demonstrating promising potential in treating neurodegenerative disorders.^205^ However, the inability of these factors to penetrate the blood‐brain barrier necessitates direct injection or the development of small molecules with similar efficacy, thereby imposing limitations on their clinical application. Given the central role of caspases in apoptosis, it is plausible to impede apoptotic processes initiated by caspase activation, thereby providing a neuroprotective effect. For instance, administration of platycodin D in mice resulted in reduced cleavage of caspase‐9 and caspase‐3, along with decreased expression levels of the proapoptotic protein Bax and increased expression of Bcl‐2.^206^ S‐nitrosylation of XIAP directly influences its anticaspase‐3 and antiapoptotic functions, underscoring the significance of XIAP in the pathogenesis of PD. Consequently, endogenous inhibitors that prevent caspase activation present a viable therapeutic option.^207^ The accumulation of ROS in lipids is a primary hallmark of ferroptosis, which is closely associated with oxidative stress. Additionally, iron plays a pivotal role in the survival and differentiation of nerve cells, contributing to the normal functioning of the nervous system.^208^ Research findings indicate that elevated levels of iron in the brain can trigger ferroptosis and potentially accelerate the progression of neurodegenerative disorders.^209^, ^210^ The efficacy of an increasing number of drugs in treating neurological disorders is being explored through the inhibition of ferroptosis.^211^ The ferroptosis inhibitor GPX4 holds significant promise as a therapeutic target for neurodegeneration. Selenium levels are critical for the activity of GPX4, and studies have shown that selenium inhibits the transcription of β‐secretase induced by the lipid peroxidation product 4‐hydroxynonenal, thereby reducing Aβ production.^212^ While the potential significance of necroptosis in neurodegeneration may have been overlooked, evidence suggests that necroptosis activation occurs during the advanced stages of AD.^213^ Consequently, common inhibitors of necroptosis may serve as alternative therapeutic options. In murine models, nec‐1 has demonstrated efficacy in attenuating neuronal cell death, reducing Aβ levels, and inhibiting the formation of neurofibrillary tangles in the brain.^214^ Similarly, the MLKL inhibitor necrosulfonamide has been shown to alleviate neuropathy in AD by specifically targeting the MLKL‐dependent necroptosis pathway.^215^ The protective effect of autophagy differs fundamentally from that of other PCD modalities. Therefore, neurodegenerative treatment strategies typically focus on inhibiting these modes of cell death, representing a clear direction for the further development of novel therapeutic approaches.

### 4.3 Autoimmune disorders

The primary physiological role of the immune system is to defend against foreign pathogens while distinguishing them from normal autoantigens. Loss of self‐tolerance can lead to inappropriate immune activation, resulting in tissue damage and autoimmune diseases. A hallmark of these disorders is the presence of autoreactive lymphocytes that target “self” molecules, causing their destruction. PCD is essential for maintaining the quantitative balance and normal function of immune cells, a concept supported by numerous studies. This summary focuses on the role of PCD in autoimmunity.

In the context of immune regulation, our research emphasizes the critical role of apoptosis in the maturation and homeostasis of the immune system. Defects in the apoptotic pathway significantly contribute to aberrant immune responses against autoantigens. Evidence indicates that defective apoptosis is associated with autoimmune diseases, with autoimmune lymphoproliferative syndrome being a prototypical example triggered by insufficient apoptosis of immune cells.^216^ Similar manifestations are observed in autoimmune and lymphoproliferative diseases resulting from FAS deficiencies.^217^ Interestingly, excessive induction of apoptosis can also contribute to the development of autoimmune disorders. During apoptosis, alterations in both plasma membrane and intracellular components may confer new immunogenicity to apoptotic cells. For instance, the rearrangement of phospholipids in the cell membrane enhances its binding capacity, facilitating the formation of novel complexes that can augment the immunogenicity of the cell surface and trigger autoimmune responses and autoantibody production.^218^ In CTL‐induced apoptosis, the proteolytic cleavage of granzyme B generates novel immunogenic peptides, thereby intensifying the autoimmune response.^219^ In patients with systemic lupus erythematosus (SLE), there is an increased frequency of CD8+ lymphocytes activated by granzyme B, leading to the production of a substantial amount of soluble nucleosomes and granzyme B‐specific fragments derived from the autoantigen U1‐70k.^220^ Another mechanism by which apoptotic cells acquire immunogenicity involves the redistribution of autoantigens, typically sequestered within cells, onto the surface of apoptotic cells. While the clearance of apoptotic cells by phagocytes, such as macrophages, is generally rapid, any impairment or delay in this clearance increases the likelihood of immune cells recognizing and responding to novel autoantigens. Molecules facilitating the connection between phagocytes and cleared cells include MFG‐E8, annexin I, protein S, and Gas6 on the surface of apoptotic cells, as well as the Mer, Axl, and Tyro3 receptors on phagocytes.^221^ In a murine model of autoimmune diabetes, deficiency in MFG‐E8 accelerates disease onset by delaying the clearance of apoptotic cells and enhancing autoantigen presentation.^222^ Additionally, the absence of the membrane tyrosine kinase c‐Mer in mice has been shown to result in phagocytosis deficiencies and the development of progressive lupus‐like autoimmunity.^223^ These examples clearly illustrate the involvement of apoptotic dysregulation in autoimmune diseases.

As a form of PCD, autophagy exhibits a distinctive cytoprotective effect, and its dualistic nature complicates its role in autoimmunity. While inhibiting autophagy has demonstrated potential for ameliorating conditions such as multiple sclerosis, SLE, and rheumatoid arthritis, it seems to exacerbate conditions like psoriasis, psoriatic arthritis, and Crohn's disease.^224^ The impact of excessive pyroptosis on autoimmune diseases is more pronounced.^225^ Numerous studies have focused on pyroptosis, particularly the NLRP3 inflammasomes as central components. Inhibiting the activation of the NLRP3 inflammasome, suppressing the release of proinflammatory cytokines, and blocking pyroptosis can effectively alleviate autoimmune diseases such as lupus nephritis, rheumatoid arthritis, and inflammatory bowel disease.^226^, ^227^, ^228^ However, the molecular mechanisms underlying the exacerbation of autoimmune diseases due to overactivated pyroptosis require further investigation. Pyroptosis, as a highly proinflammatory form of PCD, undeniably promotes dysregulated inflammation in autoimmune diseases. Ferroptosis has been observed in both immune and nonimmune cells among patients with SLE, rheumatoid arthritis, and inflammatory bowel disease, alongside apoptosis and pyroptosis.^229^ In contrast, most research has predominantly focused on elucidating the role of ferroptosis in cancer, leaving a gap in understanding its involvement and underlying mechanisms in autoimmune diseases. The absence of endogenous ferroptosis inhibitors is believed to correlate closely with the exacerbation of autoimmune conditions. Reduced expression of GPX4 mRNA in the gray matter of patients with multiple sclerosis and decreased GPX4 protein levels in mice with autoimmune encephalomyelitis have been convincingly demonstrated.^230^

Treatments targeting PCD processes are increasingly showing promise in improving autoimmune diseases. The B‐cell‐activating factor of the TNF family (BAFF), essential for B cell survival and maturation, is found at elevated levels in certain autoimmune conditions. Excessive BAFF provides additional survival signals to B cells, preventing their apoptosis. Belimumab, a human monoclonal antibody, specifically binds to soluble BAFF, interfering with normal B cell development and promoting negative selection of activated autoreactive B cells in patients with SLE.^231^ Rituximab, a chimeric monoclonal antibody directed at CD20‐positive B lymphocytes, also facilitates B cell depletion by inducing apoptosis.^232^ The combination of rituximab and intravenous immunoglobulin has proven effective in reversing autoimmunity.^233^ Apoptosis‐based B cell depletion therapy exemplifies the therapeutic strategies targeting PCD processes. Additionally, gene delivery involving apoptosis‐related genes has shown promising results in alleviating symptoms of certain autoimmune diseases. For instance, delivery of the FasL gene in autoimmune arthritis inhibits proinflammatory cytokine production and induces widespread apoptosis of synovial cells.^234^ While apoptosis‐based strategies currently appear to be the most viable approach, research on autoimmune disease treatment predominantly emphasizes immunological aspects without extensive integration with PCD. To advance more effective treatments, it is imperative to elucidate the roles of various forms of PCD in autoimmunity.

## 5 THERAPEUTIC TARGETS AND STRATEGIES

**FIGURE 4:**
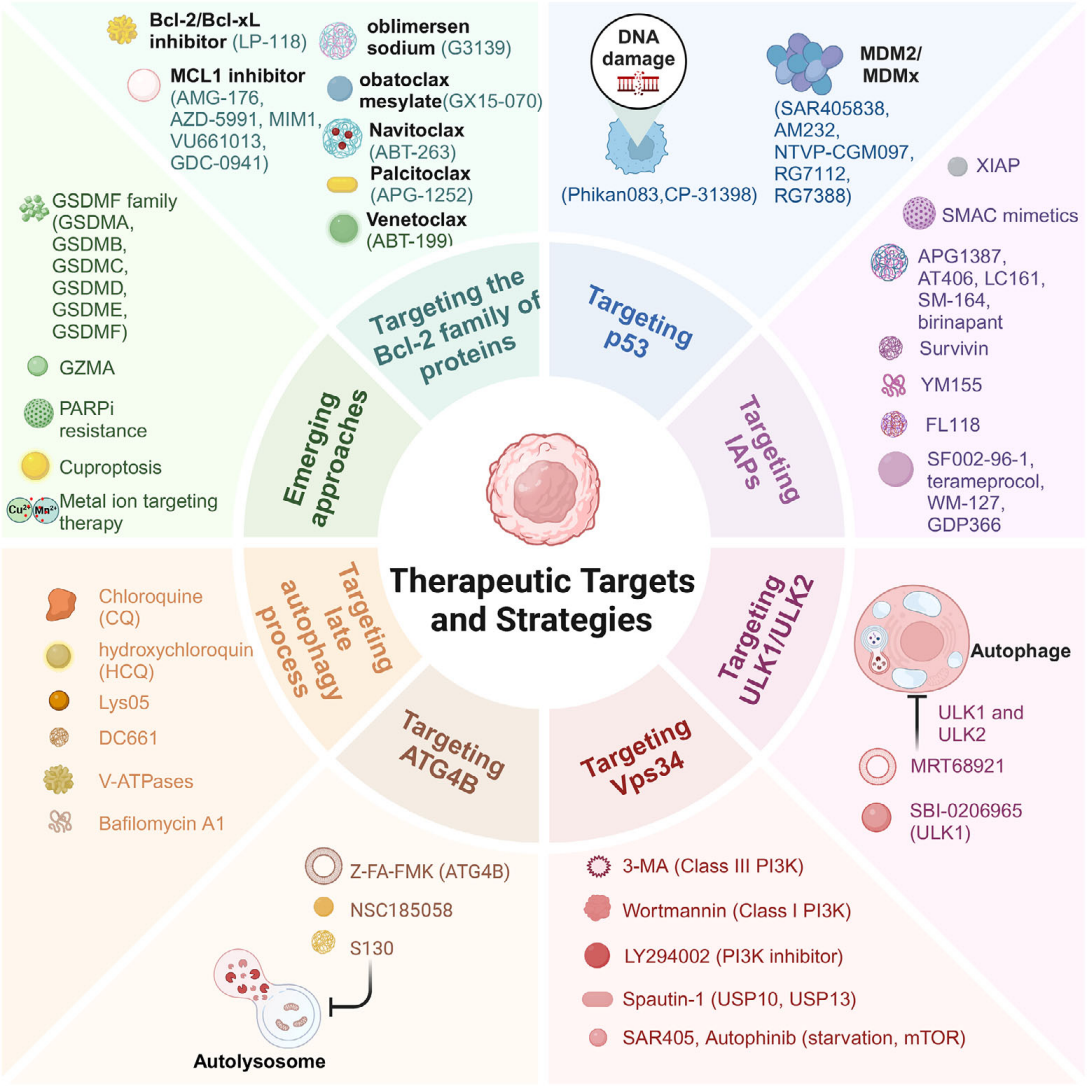
Therapeutic targets and strategies. Therapeutic strategies targeting programmed cell death focus on apoptosis (including the Bcl‐2 family of proteins, p53, and IAPs) and autophagy (targeting ULK1/ULK2, Vps34, ATG4B, and late autophagic processes). This section summarizes the regulatory factors involved and outlines small molecule drugs at various preclinical stages or those that have received approval for specific diseases. Based on current research progress, emerging methodologies, and prospective treatment avenues, this review proposes innovative therapeutic strategies. Figures were created with biorender.com.

PCD presents promising therapeutic targets and strategies for the treatment of various diseases. Pharmacological modulation serves as a key approach, wherein drugs are designed to manipulate specific molecules and pathways involved in cell death processes. Inhibitors or activators of apoptotic proteins are particularly noteworthy due to the extensive research conducted on apoptosis. Additionally, emerging strategies are making significant advancements, including gene therapy and targeted delivery of therapeutic agents to specific cells, which are paving new avenues for treatment. The application of nanoparticles and immunotherapies that engage with PCD mechanisms also demonstrates substantial potential. Ongoing research in this domain promises to transform the treatment landscape for numerous challenging diseases (Figure 4).

### 5.1 Pharmacological modulation of cell death pathways

#### 5.1.1 Small molecules targeting apoptotic regulators

Various small‐molecule drugs targeting regulators of apoptosis have been developed by different entities, with some progressing through various preclinical stages or receiving approvals for the treatment of specific diseases. This section presents a comprehensive compilation of these small molecules.

##### Targeting the Bcl‐2 family of proteins

Oblimersen sodium (G3139), the first drug to undergo clinical trials against Bcl‐2, is regarded as a classic in this field. It specifically binds to human Bcl‐2 mRNA, facilitating its catalytic degradation and subsequently reducing Bcl‐2 protein translation.^235^ In contrast to other highly selective Bcl‐2 inhibitors, obatoclax mesylate (GX15‐070) is classified as a pan‐Bcl‐2 inhibitor, capable of targeting all antiapoptotic Bcl‐2 family proteins.^236^ Navitoclax (ABT‐263) is a potent inhibitor of the Bcl‐2 family, demonstrating high affinity for Bcl‐2, Bcl‐W, and Bcl‐xL; ABT‐737 exhibits similar properties.^237^ Palcitoclax (APG‐1252) is another highly potent antagonist of the Bcl‐2 family that, like navitoclax, primarily targets Bcl‐2 and Bcl‐xL.^238^ Venetoclax (ABT‐199) is notable as the first US FDA‐approved Bcl‐2 inhibitor, exhibiting high selectivity for Bcl‐2.^7^ The oral selective Bcl‐2/Bcl‐xL inhibitor LP‐118 is distinguished by its minimal regulation of anti‐Bcl‐xL activity, effectively reducing the risk of thrombocytopenia. Among these small molecules exists a distinct class of drugs known as BH3 mimetics, which mimic the binding process between BH3‐only proteins and antiapoptotic proteins within the Bcl‐2 family.^239^ MCL1, recognized for its antiapoptotic properties, is frequently targeted as well. AMG‐176 is the first selective MCL1 inhibitor to undergo human studies, while other MCL1 inhibitors include AZD‐5991, MIM1, VU661013, and GDC‐0941.^240^

##### Targeting p53

The wild‐type p53 gene has the ability to induce apoptosis, with the encoded p53 protein serving as a checkpoint in the G1 phase of the cell cycle to assess potential chromosomal DNA damage. In cases where DNA repair fails, p53 initiates apoptosis.^241^ Several small molecules can restore the wild‐type functionality of mutated p53. Phikan083 exhibits binding affinity and restoration potential for mutant p53, while CP‐31398 can intercalate DNA, modify, and disrupt the DNA–p53 core domain complex, leading to the recovery of unstable p53 mutants.^242^, ^243^ Murine double minute 2 (MDM2) and MDMX act as prominent negative regulators of p53, with numerous small molecules developed to specifically target the interaction between MDM2/MDMX and p53. RG7112 was the first imidazole MDM2 small molecule inhibitor to enter clinical trials, followed by RG7388, synthesized as a class of pyrrolidine derivatives. Other MDM2–p53 inhibitors include AM232, SAR405838, and NTVP‐CGM097.^244^ However, the binding affinity of many small molecules targeting MDM2 for MDMX is limited, and currently, there are no specific inhibitors available for MDMX.

##### Targeting the IAPs

XIAP is recognized as the most potent inhibitor of apoptosis among all identified IAPs. Following MOMP, the release of SMAC is essential in preventing direct inhibition of caspase‐3, ‐7, and ‐9 by XIAP. To address this, specific small molecules known as SMAC mimetics have been developed, with several compounds identified, including APG1387, AT‐406, LC161, SM‐164, and birinapant.^245^ Survivin, a newer member of the IAP family, is targeted by the small molecule YM155, which significantly inhibits survivin expression at both mRNA and protein levels.^246^ The compound FL118 is a nonselective inhibitor of survivin expression, effectively suppressing the activity of the survivin promoter.^247^ Other survivin inhibitors include SF002‐96‐1, terameprocol, WM‐127, and GDP366^248^ (Table 1).

**TABLE 1:** Clinical trials of apoptosis inhibitors.

Apoptosis inhibitor	Target	Condition and disease	Number of included patients	Trail number	Phase
Genasense (G3139)	Bcl‐2	Melanoma	7	NCT01200342	II
Genasense (G3139)	Bcl‐2	Diffuse large B‐cell lymphoma	37	NCT00736450	I
Genasense (G3139)	Bcl‐2	Solid tumors	25	NCT00636545	I
Obatoclax mesylate (GX15‐070)	Bcl‐2	Leukemia	50	NCT01150656	NA
Obatoclax mesylate (GX15‐070)	Bcl‐2	Leukemia, systemic mastocytosis	3	NCT00918931	II
Navitoclax (ABT‐263)	Bcl‐2	Myeloid neoplasms	16	NCT05455294	I
Navitoclax (ABT‐263)	Bcl‐2	Acute myeloid leukemia	17	NCT05222984	I
ABT‐737	Bcl‐2	Ovarian cancer	36	NCT01440504	NA
Pelcitoclax (APG‐1252)	Bcl‐2	Small cell lung cancer, solid tumor	50	NCT03080311	I
Venetoclax (ABT‐199)	Bcl‐2	Acute myeloid leukemia	500	NCT06429670	NA
Venetoclax (ABT‐199)	Bcl‐2	Idiopathic pulmonary fibrosis	3	NCT05976217	I
Venetoclax (ABT‐199)	Bcl‐2	Chronic lymphocytic leukemia	100	NCT05555979	NA
AMG‐176	MCL1	Chronic myelomonocytic leukemia	7	NCT05209152	I
AMG‐176	MCL1	Relapsed or refractory multiple myeloma, relapsed or refractory acute myeloid leukemia	142	NCT02675452	I
Pictilisib (GDC‐0941)	MCL1	Breast cancer	183	NCT01740336	II
Pictilisib (GDC‐0941)	MCL1	Non‐small cell lung cancer	501	NCT01493843	II
RO5045337 (RG7112)	MDM2–p53	Hematologic neoplasms	116	NCT00623870	I
RO5045337 (RG7112)	MDM2–p53	Neoplasms	106	NCT00559533	I
Idasanutlin (RG7388)	MDM2–p53	Acute myeloid leukemia, acute lymphoblastic leukemia, neuroblastoma, solid tumors	38	NCT04029688	I/II
Idasanutlin (RG7388)	MDM2–p53	Solid tumors	48	NCT03362723	II
Idasanutlin (RG7388)	MDM2–p53	Leukemia, myeloid	88	NCT02670044	I
SAR405838	MDM2–p53	Neoplasm malignant	26	NCT01985191	I
SAR405838	MDM2–p53	Neoplasm malignant	77	NCT01636479	I
APG1387	SMAC	Advanced solid tumors or hematologic malignancies	90	NCT03386526	I
Debio 1143 (AT‐406)	SMAC	Solid tumor, lymphoma	51	NCT01078649	I
Birinapant (TL32711)	SMAC	Relapsed ovarian cancer	27	NCT01940172	I
Birinapant (TL32711)	SMAC	Solid tumor	176	NCT01188499	I/II
Birinapant (TL32711)	SMAC	Refractory solid tumor, refractory lymphoma	50	NCT00993239	I
YM155	Survivin	Non‐small cell lung cancer, solid tumor	42	NCT01100931	I/II
YM155	Survivin	Breast cancer	101	NCT01038804	II
YM155	Survivin	Melanoma	64	NCT01009775	II
Terameprocol (EM‐1421)	Survivin	High grade glioma	20	NCT02575794	I
Terameprocol (EM‐1421)	Survivin	Brain and central nervous system tumors	35	NCT00404248	I/II
Terameprocol (EM‐1421)	Survivin	Cervical intraepithelial neoplasia	8	NCT00154089	I/II

*Data source*: https://clinicaltrials.gov/.

Although numerous regulators are involved in the apoptosis pathway with diverse roles, the majority of relevant small molecules primarily target the aforementioned three components. Continued research and innovative approaches are necessary to expand the repertoire of small molecules specifically targeting apoptotic regulators.

#### 5.1.2 Modulation of autophagy for therapeutic purposes

The complexity of autophagy's dual role in pathogenesis is increasingly recognized, with its protective or destructive nature contingent upon the specific disease and cell type involved. The subsequent section will provide a comprehensive overview of the extensively studied upregulation of autophagy in the treatment of neurodegenerative diseases. Emerging evidence suggests that cell‐protective autophagy can contribute to disease progression, such as tumorigenesis, highlighting the critical importance of investigating autophagy inhibitors. This section will summarize these inhibitors.

##### Targeting ULK1/ULK2

Utilizing modulators that target ULK1 and ULK2 to impede the initial stages of autophagy represents a highly effective strategy for inhibiting this cellular process. Early ULK inhibitors included MRT68921 and SBI‐0206965. MRT68921 demonstrates selective inhibition of both ULK1 and ULK2; however, it also exhibits a high affinity for tank‐binding kinases, thereby inhibiting their activity.^249^ SBI‐0206965 is a highly selective ULK1 kinase inhibitor in vitro, effectively suppressing ULK1‐mediated phosphorylation events in cellular systems.^250^ The efficiency and selectivity of ULK‐101 surpass those of MRT68921 and SBI‐0206965.^251^

##### Targeting Vps34

Vps34, a unique member of the Class III PI3K family, plays a pivotal role in autophagy activation, and numerous inhibitors targeting Vps34 have been developed. Among these, 3‐MA stands out as the most extensively utilized autophagy inhibitor. While it targets Vps34, it also exhibits inhibitory effects on Class I PI3K activity, thus exerting a dual impact.^252^ Wortmannin, a mold metabolite, exerts a durable effect on Vps34 and transient inhibition on Class I PI3K, making it a more suitable candidate than 3‐MA as an autophagy inhibitor.^253^ LY294002 was the first synthetic PI3K inhibitor, while LY204992 is considered more selective but is generally regarded as nonspecific.^254^ The precise effects of these Vps34 inhibitors on autophagy remain unclear, necessitating improved specificity for Vps34 compounds and exploration of new Vps34 inhibitors. Spautin‐1 is a specific and potent autophagy inhibitor that targets ubiquitin‐specific peptidases USP10 and USP13, resulting in the breakdown of the Vps34 complex.^255^ SAR405 can inhibit starvation and mTOR inhibitor AZD8055, both of which induce autophagy. Autophinib, similar to SAR405, is a selective Vps34 inhibitor.^256^

##### Targeting ATG4B

During the extension phase of autophagy, ATG4B cleaves ATG8, making it a potential target for autophagy regulation. Z‐FA‐FMK is a covalent inhibitor of ATG4B that also blocks cathepsin B and calpain.^257^ NSC185058 has been shown to inhibit autophagy by decreasing ATG4B levels, without affecting mTOR and PI3K activity.^258^ S130 is a newly discovered inhibitor with a high affinity for ATG4B, potentially attenuating the delipidation of LC3‐II on autolysosomes and suppressing the recycling of LC3‐I.^259^

##### Targeting late autophagy process

The fusion and degradation stages occur during the late phase of autophagy and serve as additional targets for regulating the autophagic process. CQ and hydroxychloroquine (HCQ) primarily inhibit degradative enzyme activity by elevating lysosomal pH, preventing lysosomal digestion and effectively inhibiting autophagy.^260^ This class of drugs is referred to as lysosomotropic agents. Lys05, a bivalent aminoquinoline analog of HCQ, is more likely to accumulate in lysosomes, resulting in a significant pH increase.^261^ DC661, another CQ derivative, targets palmitoyl‐protein thioesterase 1 (PPT1), with PPT1 deficiency being a contributor to lysosomal dysfunction^262^ Additionally, V‐ATPase inhibitors are effective autophagy inhibitors, as V‐ATPases are responsible for lysosomal acidification, which stimulates the activity of resident hydrolases. Bafilomycin A1 is a representative drug in this category; it blocks lysosomal transport by inhibiting V‐ATPases, thus inhibiting lysosomal hydrolase activity^263^ (Table 2).

**TABLE 2:** Clinical trials of autophagy inhibitors.

Autophagy inhibitor	Target	Condition and disease	Number of included patients	Trail number	Phase
Chloroquine (CQ)	Degradative enzymes	Glioblastoma multiforme	13	NCT02378532	I
Chloroquine (CQ)	Degradative enzymes	Hepatitis C virus	10	NCT02058173	IV
Chloroquine (CQ)	Degradative enzymes	Ductal carcinoma in situ	12	NCT01023477	I/II
Chloroquine (CQ)	Degradative enzymes	Pancreatic cancer	9	NCT01777477	I
Chloroquine (CQ)	Degradative enzymes	Small cell lung cancer	5	NCT00969306	I
Hydroxychloroquine (HCQ)	Degradative enzymes	Metastatic melanoma	31	NCT04760080	NA
Hydroxychloroquine (HCQ)	Degradative enzymes	Pancreatic cancer	52	NCT04386057	II
Hydroxychloroquine (HCQ)	Degradative enzymes	Estrogen receptor positive, HER2 negative breast cancer	15	NCT03774472	I/II
Hydroxychloroquine (HCQ)	Degradative enzymes	Cholangiocarcinoma	65	NCT03377179	II
Hydroxychloroquine (HCQ)	Degradative enzymes	Colorectal cancer	42	NCT02316340	II
Hydroxychloroquine (HCQ)	Degradative enzymes	Advanced BRAF mutant melanoma	50	NCT02257424	I/II
Hydroxychloroquine (HCQ)	Degradative enzymes	Lymphangioleiomyomatosis	14	NCT01687179	I
Hydroxychloroquine (HCQ)	Degradative enzymes	Non‐small cell lung cancer	32	NCT01649947	II
Hydroxychloroquine (HCQ)	Degradative enzymes	Metastatic clear cell renal cell carcinoma	40	NCT01510119	I/II
Hydroxychloroquine (HCQ)	Degradative enzymes	Pancreatic cancer	119	NCT01506973	I/II
Hydroxychloroquine (HCQ)	Degradative enzymes	Advanced cancers	143	NCT01266057	I
Hydroxychloroquine (HCQ)	Degradative enzymes	Colorectal cancer	50	NCT01206530	I/II
Hydroxychloroquine (HCQ)	Degradative enzymes	Malignant solid tumor	72	NCT01023737	I
Hydroxychloroquine (HCQ)	Degradative enzymes	Colorectal cancer	38	NCT01006369	II

*Data source*: https://clinicaltrials.gov/.

### 5.2 Targeted therapies for specific diseases

#### 5.2.1 Apoptosis‐targeting therapies for cancers

The identification of defects or abnormalities in apoptosis presents promising targets for cancer therapy. Therapeutic strategies or drugs capable of restoring normal function to apoptosis signaling pathways hold significant potential for eliminating cancer cells that evade apoptosis for survival. This review focuses on apoptosis‐targeting therapies for cancer.

Initial efforts primarily concentrated on inhibiting antiapoptotic proteins within the Bcl‐2 family. Small molecule inhibitors targeting Bcl‐2 have demonstrated superior efficacy across various cancer types. Numerous studies have shown that combining G3139 with chemotherapy results in a substantial enhancement of antitumor responses and increased tumor cell apoptosis in both small‐cell lung cancer and NSCLC.^264^ Venetoclax has been effectively employed in the treatment of acute myeloid leukemia (AML), chronic lymphocytic leukemia (CLL), and mantle cell lymphoma (MCL).^7^, ^265^ Its unique targeting and highly effective antitumor effects have shown significant promise in the field of blood cancer therapy. ABT‐737 has also demonstrated effectiveness against lymphomas, leukemias, and solid tumors; however, resistance to its death‐inducing effects has been reported, necessitating further investigation into the underlying mechanisms. The MCL1 inhibitor AMG‐176 has shown efficacy in inducing apoptosis in CLL while preserving the viability of normal hematopoietic cells.^266^ Additionally, AMG‐176 and venetoclax exhibit a synergistic effect in AML models.^267^ Beyond pharmacological interventions, apoptosis can also be upregulated through gene silencing targeting the Bcl‐2 antiapoptotic protein family. For instance, both in vivo and in vitro experiments have demonstrated that exosome‐coated Bcl‐2 siRNA effectively penetrates tumors within the digestive system, leading to tumor growth inhibition^268^ New methods for transporting Bcl‐2 siRNA continue to emerge. A carrier, DPL, developed from diketopyrrolopyrrole (DPP), can facilitate targeted delivery of Bcl‐2 siRNA and enable tumor imaging both in vitro and in vivo.^269^

Utilizing p53‐based gene therapy may also be a viable option for tumor treatment.^270^ The p53 protein plays a vital role in cellular sensitivity and resistance to antitumor drugs, with p53‐induced apoptosis enhancing tumor resistance to chemotherapy.^271^ Additionally, mutated p53 expression often leads to tumor radioresistance.^272^ Introducing the wild‐type p53 gene has been shown to increase the susceptibility of tumor cells in head and neck cancers, colorectal cancer, prostate cancer, and gliomas to ionizing radiation.^273^ Tumor suppressor p53 (TP53) is the most frequently mutated gene in tumors, with these mutations resulting in both loss of function and gain‐of‐function alterations that promote tumor progression and metastasis. Consequently, p53 represents a promising target for cancer therapy. Potential therapeutic strategies focusing on p53 primarily involve activating wild‐type p53, reactivating mutant p53, or eliminating cells harboring p53 mutations.

IAP represents an attractive molecular target for developing new strategies to combat cancer. IAP antagonists have shown significant promise in treating head and neck cancer, particularly in combination with radiation therapy.^274^ For instance, the SMAC mimetic APG‐1387 demonstrated antinasopharyngeal carcinoma activity at well‐tolerated doses, significantly downregulating cell viability in nasopharyngeal carcinoma.^275^ Furthermore, the antitumor efficacy of APG‐1387 has also been confirmed in hepatocellular carcinoma.^276^ Another SMAC analog, LCL‐161, exhibited potent antitumor activity in a preclinical model of rituximab‐resistant B‐cell lymphoma.^277^ In addition to small molecules, oncology therapies targeting IAPs include antisense oligonucleotides and siRNA. Downregulation of XIAP through antisense oligonucleotides has been shown to inhibit the growth of human NSCLC both in vivo and in vitro.^278^ Moreover, knocking down IAP genes with siRNA can synergistically inhibit the proliferation and transformation abilities of high‐grade bladder cancer T24 cells, while enhancing their apoptotic sensitivity to chemotherapy.^279^ Therefore, silencing IAP genes may emerge as an effective gene therapy approach.

The caspase hydrolysis cascade, which is essential for the final execution of apoptosis, must not be overlooked in tumor therapies targeting this process. For example, Junduqing extract promotes cell apoptosis by upregulating caspase‐3, caspase‐8, and caspase‐9, resulting in inhibited proliferation, migration, and invasion of human NPC cells.^280^ Additionally, the caspase inducer Apoptin, originally derived from the chicken anemia virus, selectively triggers apoptosis in malignant cells while sparing nonmalignant cells.^281^

Given the complexity and delicacy of the apoptosis process, both the identified apoptotic targets and the yet‐to‐be‐discovered regulatory pathways may serve as entry points for innovative tumor treatments. Thus, this area of research presents a vast and enticing opportunity for advancements in cancer therapy.

#### 5.2.2 Autophagy‐targeted treatments for neurodegenerative diseases

Autophagy dysfunction has been implicated in the pathogenesis of neurodegenerative diseases, providing a strong theoretical foundation for upregulating autophagy as a therapeutic approach. The promotion of autophagy is primarily achieved through the use of autophagy inducers, which can be broadly categorized into two groups: mTOR‐dependent and mTOR‐independent inducers. mTOR inhibitors are theoretically well suited for this purpose, as they can inhibit mTOR through both competitive and noncompetitive mechanisms. ATP‐competitive inhibitors include Torin1, while noncompetitive inhibitors encompass Rapamycin and its derivatives, known as Rapalogs.^282^ ATP‐competitive mTOR inhibitors effectively inhibit both mTORC1 and mTORC2 and may also affect PI3K activity, raising concerns about potential toxicity.^283^ Nonetheless, studies have indicated that Torin1 treatment upregulates lysosomal biogenesis and enhances autophagic clearance in Gaucher's disease neurons, a condition where neurodegeneration is a prominent manifestation.^284^ Conversely, the safety profile of Rapamycin and Rapalogs is enhanced due to their non‐ATP‐competitive nature. Emerging evidence suggests that Rapamycin exerts therapeutic effects on various neurodegenerative disorders, including AD, PD, Huntington's disease, FTD, and amyotrophic lateral sclerosis.^285^ Additionally, Rapalogs offer advantages such as improved tolerability and micro‐delivery modes.

Most mTOR‐independent autophagy inducers operate through the AMPK pathway. Trehalose is one such drug that has been extensively studied. It targets GLUT proteins, leading to their inhibition and subsequent activation of AMPK.^286^ The administration of trehalose has been shown to induce autophagy in various classical neurodegenerative models, demonstrating efficacy due to its natural occurrence as a disaccharide, resulting in minimal side effects.^287^ Furthermore, trehalose enhances autophagy via transcription factor EB and ameliorates disease phenotypes in various models of neurodegenerative disorders.^288^ It has also been effective in reducing memory and behavioral disorders, as well as neuroinflammatory processes in the brain, making it a promising therapeutic agent with potential for further refinement.^289^ Metformin, another AMPK‐dependent autophagy inducer, has also shown beneficial effects. In a paraquat‐induced PD model, metformin and trehalose were found to induce autophagy through AMPK phosphorylation, significantly reducing PD phenotypes.^290^ Additionally, crocetin has been shown to induce autophagy by activating AMPK, demonstrating efficacy in models of AD.^291^ Other compounds such as berberine, methylene blue, and nilotinib exhibit similar properties.^292^, ^293^, ^294^ In summary, upregulating autophagy is a common goal across various approaches to the treatment of neurodegeneration.

Loss of neuronal CMA results in significant alterations in both the quantity and quality of the neuronal proteome, ultimately leading to the degeneration of critical neuronal functions. Increasing evidence suggests that CMA activity is diminished in conditions such as PD, FTD, and AD, rendering CMA an attractive target for therapeutic intervention in neurodegenerative diseases.^295^

Prolyl oligopeptidase (PREP) may serve as a central node linking macrophages and CMA. The small molecule inhibitor KYP‐2047 has been shown to enhance the degradation of α‐synuclein by accelerating macrophage activity. Additionally, inhibition of PREP can lower α‐synuclein levels at the CMA level.^296^ Since retinoic acid receptor alpha negatively regulates CMA, retinoic acid derivatives can activate this pathway.^297^ Optimized derivatives, such as CA77.1, have demonstrated enhanced efficacy in alleviating tau and Aβ‐related pathologies in mouse models of FTD.^194^ The regulation of CMA in neurodegenerative diseases has focused on LAMP2A. Damage to cerebellar neuronal CMA induced by LAMP2A knockout can lead to ataxia and neurodegeneration.^298^ Conversely, overexpression of LAMP2A has been shown to effectively reduce levels of α‐synuclein and associated abnormal inclusion bodies, thereby mitigating neurodegeneration^299^ Furthermore, inhibition of HDAC6 by Tubastatin A has been reported to reduce pathology associated with PD through the activation of CMA, resulting in increased levels of LAMP2A.^300^ Despite the current absence of established means or small molecules specifically targeting LAMP2A, its therapeutic potential remains promising. All substrates of CMA possess a KFERQ‐like motif recognized by HSC70, characterized by the amino acid sequence Lys–Phe–Glu–Arg–Gln. One study successfully designed peptides containing the KFERQ motif to bind Aβ polymers and facilitate their degradation via CMA, demonstrating the feasibility of this approach.^301^ Thus, utilizing the KFERQ motif to label target polymeric proteins appears to be a viable strategy for CMA‐based therapeutic interventions.

### 5.3 Emerging approaches and future directions

PCD is an exquisitely delicate physiological process, and despite decades of research since the discovery of apoptosis, our understanding remains only superficial. With advances in molecular technology, new therapeutic targets within the realm of PCD are continually emerging, some of which have demonstrated efficacy in in vivo or in vitro experiments, while others are still being conceptualized. The development of therapeutic strategies based on diverse targets represents a significant progression in precision medicine, highlighting a notable trend toward greater selectivity in newly developed small molecules. This synergy illustrates that PCD and precision medicine are not mutually exclusive; rather, they complement each other.

Historically, research on pyroptosis has primarily concentrated on GSDMD. However, humans possess six gasdermin family members: GSDMA, GSDMB, GSDMC, GSDMD, GSDME, and GSDMF.^302^ Except for GSDMF, all gasdermins feature a conserved pore formation domain at their N‐terminal regions, thus presenting them as potential therapeutic targets in pyroptosis. GSDMA, which is the principal gasdermin in the skin, has been implicated in pyroptosis triggered by streptococcal pyrogenic exotoxin B, which cleaves GSDMA. Deficiencies in the GSDMA1 gene have been linked to uncontrolled bacterial dissemination and increased mortality in mice, suggesting that targeting GSDMA could offer a promising therapeutic approach for infections caused by Streptococcus pyogenes.^303^ GSDMB levels are elevated in inflammatory bowel disease, with GSDMB‐dependent pyroptosis playing a critical role in restoring epithelial barrier function and resolving inflammation^304^ Additionally, granzyme A (GZMA), traditionally associated with apoptosis, has been found to cleave GSDMB, facilitating tumor clearance when GZMA‐cleavable GSDMB is introduced into mouse cancer cells.^305^ Furthermore, resistance to PARP inhibitors (PARPi) is commonly observed in tumors; however, GSDMC/Caspase‐8‐mediated pyroptosis can enhance the cytotoxic effects of PARPi on tumor cells, presenting a novel approach to overcoming PARPi therapeutic resistance.^306^

Cuproptosis, a newly identified form of PCD following ferroptosis, emphasizes the importance of copper homeostasis and various cuproptosis‐related proteins. Disruptions in copper levels can lead to adverse health effects, including mitochondrial dysfunction, Wilson's disease, neurodegenerative diseases, cancer, and cardiovascular disorders.^95^ SLC31A1, ATP7A, and ATP7B play critical roles in regulating intracellular copper ion concentrations, with SLC31A1 exhibiting elevated expression in various cancer types, thus positioning it as a promising predictive biomarker.^307^ FDX1, a ferredoxin identified as a central molecule in cuproptosis, is modified by lactate‐mediated m6A methylation of its mRNA via METTL16, promoting cuproptosis in gastric cancer and significantly enhancing the therapeutic effect of elesclomol.^308^ Additionally, FDX1 interacts with LIAS to regulate protein lipid acylation.^308^ Recent analyses of extensive RNA‐seq datasets indicate that LIAS‐mediated cuproptosis may be involved in a comprehensive cellular and molecular mechanism contributing to the onset and progression of SLE.^309^ Several cuproptosis‐related genes, including FDX1 and LIAS, have been identified by Tsvetkov et al.^93^ Although the mechanisms of cuproptosis are not yet fully elucidated, a growing body of research supports the feasibility of targeting these pathways for therapeutic purposes.

Metal ion‐targeting therapy has emerged as a prominent area of research. The discovery of calcicoptosis occurred somewhat serendipitously; computed tomography findings from tumors undergoing radiotherapy and chemotherapy revealed calcified spots within tumor lesions, suggesting a connection between calcium (Ca) levels and treatment efficacy.^310^ Intracellular oxidative stress induced by these therapies disrupts the strict regulation of Ca^2+^ concentration within cells, leading to Ca overload that results in tumor cell death and calcifications.^311^ This phenomenon has been termed calcicoptosis. Building on this concept, Wenbo skillfully employed ultra‐small Ca peroxide to activate tumor cells, generating a unique biological effect characterized by “Ca overload.” This innovation led to the proposal of a new therapeutic strategy based on calcicoptosis, termed ion interference therapy.^312^ Research into calcicoptosis as a potential therapeutic approach for tumors is gradually gaining traction. For instance, a recent study by Guo demonstrated that purple sweet potato anthocyanins target NFAT5 to induce calcicoptosis, mediating an antileukemic effect.^313^ Although the precise mechanism of cell death resulting from Ca overload remains to be elucidated, it undoubtedly offers novel insights for tumor therapy.^314^

The concept of PCD aligns closely with precision medicine, reflecting the direction of contemporary medical development. An increasing body of research indicates that targeting PCD embodies the principles of precision medicine. Radiation and chemotherapy have traditionally served as powerful yet nonselective methods for eradicating cancer cells. In contrast, precision medicine revolutionizes cancer treatment by emphasizing the selective targeting of cancer cells. Poly (ADP‐ribose) polymerase (PARP) inhibitors exemplify this approach.^315^ The molecular mechanism underlying PARP‐dependent cell death involves the release of mitochondrial apoptosis‐inducing factor and its translocation to the nucleus, resulting in chromatin dissolution.^316^ Inhibition of PARP represents a primary treatment strategy for BRCA mutant ovarian cancer. Notably, pharmacological inhibition or gene deletion of PARP has been shown to downregulate the expression of the cystine transporter SLC7A11 in a p53‐dependent manner, thereby promoting lipid peroxidation and ferroptosis. PARP inhibitors can work in synergy with ferroptosis inducers to treat BRCA‐proficient ovarian cancer more precisely.^317^ Additionally, the TP53 has been shown to limit ferroptosis by inhibiting the activity of dipeptidyl‐peptidase‐4 (DPP4). The loss of p53 promotes plasma membrane‐associated DPP4‐dependent lipid peroxidation, ultimately leading to ferroptosis. This finding presents a precision medicine strategy for treating colorectal cancer by inducing ferroptosis.^318^ Furthermore, to provide new insights and tools for personalized medicine and precision therapy, the Programmed Cell Death Index was developed based on transcriptomic analysis of lung adenocarcinoma, revealing distinct cell death patterns in this cancer type.^319^

Whether through the development of new PCD targets or the heightened emphasis on selectivity, these efforts reflect the principles of precision medicine within modern healthcare. This ongoing pursuit aims to enhance human health while addressing the limitations of current treatment methodologies. Maximizing the efficacy of cell killing while preserving the physiological functions of normal tissues remains the paramount consideration in ensuring safety.

## 6 CONCLUSIONS

Cells in the human body are destined to die, making the autonomic and orderly death of cells, regulated by genes, essential for maintaining internal environmental stability. PCD, which has evolved from the initial discovery of apoptosis to the recent identification of mechanisms like cuproptosis, encompasses more than a dozen distinct forms, yet it continues to expand. The rapid advancements in molecular technology are constantly unveiling the intricate molecular mechanisms underlying PCD. Properly regulated PCD is critical for the normal development of various physiological functions, and targeting these pathways holds significant potential to profoundly influence the occurrence of numerous diseases. Despite the growing recognition of the importance of PCD in conditions such as tumors and neurodegeneration and the development of diverse protein‐targeted therapies, the effectiveness and safety of these approaches remain largely unproven, significantly limiting their clinical application. This review aims to present the molecular mechanisms underlying several major forms of PCD based on existing laboratory and clinical evidence. Furthermore, this study discusses the roles of PCD in both physiological and pathological contexts while summarizing current therapeutic strategies that target key proteins involved, providing appropriate future prospects. Ultimately, our goal is to offer valuable insights for further research into PCD.

Normal PCD is intricately involved in a variety of physiological processes throughout life. During embryonic development, PCD regulates cell quality, shapes tissues or organs, and eliminates transient structures to ensure proper development. Additionally, PCD is critical for lymphocyte selection, contributing to the establishment of the immune system and refining the body's balance of immune responses to prevent autoimmune diseases. Under various external and internal stimuli, cells inevitably encounter stress, necessitating PCD for clearance, adaptation, and survival.

As individuals age, the number of infected and damaged cells increases, highlighting the essential role of PCD in their clearance. Dysregulated PCD is thus implicated in disease development. Not only is apoptosis involved, but various forms of PCD evasion are also considered significant contributors to tumor progression, which aligns with the hallmark characteristic of uncontrolled tumor cell proliferation. Furthermore, dysregulated autophagy fosters abnormal protein aggregation, a prominent feature of neurodegenerative diseases. The loss of immune tolerance inevitably leads to the emergence of autoimmune diseases, and dysregulated PCD is frequently observed in these contexts as well.

PCD represents a critical target for a variety of therapeutic interventions aimed at addressing significant health threats, including cancer and neurodegenerative diseases. The ongoing development and optimization of small molecules remain a focal point in contemporary research. Various agonists and inhibitors can actively modulate the PCD process, enabling direct regulation of protein expression at the gene level, although this approach presents considerable challenges.

Currently, many potential targets are at the stage of serving as biomarkers for predictive applications, which, while valuable, face obstacles in terms of further basic research and clinical translation. Successful clinical implementation necessitates activation of PCD specifically within tumor cells rather than the surrounding tumor microenvironment, underscoring the importance of precision in drug delivery. Compared with normal blood vessels, tumor vasculature is characterized by incomplete structure, irregular distribution, and turbulent blood flow, which complicate the delivery of therapeutic agents into tumors.^320^ Therefore, the translation of findings from basic research into clinical applications is essential for developing novel materials that effectively address drug delivery challenges. Nanomaterials have emerged as promising carriers for therapeutic agents. However, maximizing the cytotoxic effects of PCD while preserving the physiological functions of normal tissues presents a significant challenge. Detailed investigation of regulatory targets within the human body is necessary to ensure that these targets do not interfere with other biological pathways. Additionally, ensuring the stability of various small molecules, vectors, and gene editing technologies within the body remains a critical issue, as degradation before achieving therapeutic efficacy must be prevented. The development of all medical technologies must prioritize safety, convenience, efficacy, and cost effectiveness to provide comprehensive support to patients. In conclusion, while research on PCD has made notable strides, substantial exploration remains to be undertaken.

## AUTHOR CONTRIBUTIONS

Y. T., X. Z., and Z. X. conceived the projects and revised the manuscript. S. Q. searched for literature and wrote the manuscript and summarized the table. Y. L. drew the figures. G. T., X. L., and B. X. provided their corrective comments and tips. All authors helped edit and revise the manuscript. All authors have read and approved the article and agree with publication in this journal.

## CONFLICT OF INTEREST STATEMENT

The authors declare that they have no conflict of interest.

## ETHICS STATEMENT

Not applicable.

## Data Availability

The data that support the findings of this study are available from the corresponding authors upon reasonable request.
